# BWDAT: A research tool for analyzing the consumption of VOD content at home

**DOI:** 10.1016/j.abrep.2020.100336

**Published:** 2020-12-30

**Authors:** José A. Cordeiro, Deborah Castro, Valentina Nisi, Nuno J. Nunes

**Affiliations:** a Instituto Superior Técnico de Lisboa, University of Lisbon, Portugal; b Erasmus University Rotterdam, The Netherlands & ITI/LARSyS, Portugal; c Instituto Superior Técnico de Lisboa, University of Lisbon & ITI/LARSyS, Portugal

**Keywords:** Binge-watching, Video-on-demand, Physiological data, Browser Extension, Smartwatch, Netflix

## Abstract

**Introduction:**

New approaches to the study of the binge-watching phenomenon require new technology, leading to the development of a non-intrusive and low-cost analytical research software that facilitates a holistic understanding of binge-watching in an uncontrolled environment remotely (e.g., the home). BWDAT was developed to allow the collection of three types of data: users’ physiological data gathered from a smartwatch, users’ interactions from video-on-demand interfaces, and self-reported data. This tool offers the possibility to generate automatic data analysis reports, facilitating researchers’ data analysis tasks.

**Methods:**

Two trial studies and a long-term study were used to evaluate the design and the technical implementation of the BWDAT tool. The metrics used were the BWDAT smartwatch’s App data coverage of the viewing sessions, and the data’s reliability of the viewer’s interactions with the Netflix interface, collected by the BWDAT Chrome Extension.

**Results:**

High percentages of data coverage and content coverage were verified in the sessions collecting the smartwatch’s data. The reporting system developed proved to be useful in the collection and synchronization of physiological and users’ interaction data with Netflix interface, both generated in uncontrolled environments. Furthermore, the BWDAT tool facilitated the analysis of a large amount of nuanced data.

**Conclusion:**

The results obtained confirm the reliability, accuracy, and usability of BWDAT. This tool has the potential to help researchers shed new light on the field of media and audience studies, and in particular on binge-watching.

## Highlights

• BWDAT is a reliable tool that facilitates the study of viewing experience on VOD platforms.
• Collects users’ physiological data and users’ interactions with Netflix interface.
• Non-intrusive and easy to use, successfully used in long-term projects with more than 200 users.
• Includes a graphical display of the viewing sessions to help researchers visualize the data.
• Includes an automatic report generator and data exporter for multiple platforms.

## 1 Introduction

Media consumption is changing rapidly in tandem with computing technology. The widespread integration of video-on-demand (VOD) services into consumer smart-TVs, computer applications, smart-devices apps, and pay-TV providers illustrates the increasing importance of this media distribution modality. Retaining viewers for as long as possible, engaging them in presumed addictive behaviors, and a consequent “vicious cycle” was, according to some scholars, the ultimate goal of linear television (Berger, 1978). An objective that seems to persist in the streaming era with the re-emergence and popularization of binge-watching (for an introduction to the concept see Jenner, 2015).

Broadly defined as the consumption of multiple episodes of a show in one sitting, the term binge-watching^1^ has frequently been paired to the word of “addiction” in the press (Pierce-Grove, 2016). Furthermore, and as pointed out by Flayelle et al. (2020, p. 45), there is a “widespread assumption in the literature that binge-watching has addictive qualities”. In this vein, international scholars have explored the effects that TV consumption has on viewers’ social life and health-related behaviors (Basterra-Gortari et al., 2014, Exelmansa and Van den Bulckb, 2017, Shirakawa et al., 2016, Van den Bulck, 2000). In the era of Internet Streaming Content and in the exceptional current pandemic times when people are encouraged (or even forced) to spend more time at home, this area of research is more relevant than ever. For instance, the consumption of VOD content significantly increased during the COVID-19 outbreak given the recommendation by most countries to stay at home. Data shows that the time spent consuming streaming TV and video in the weekend of March 13–14, 2020 grew by more than 40% in countries like Austria and Spain.^2^

To study binge-watching, international scholars^3^ have relied on online surveys (Riddle et al., 2017, Starosta et al., 2019) in order to better understand viewers’ motivations and habits, and physiological effects of binge-watching. Others have used interviews and focus groups (Steiner & Xu, 2018) or the combination of both (Flayelle et al., 2017, Panda and Pandey, 2017) to learn about the main binge-watching traits and college students' intentions towards binge-watching. Despite the limitations of self-reported data as a unique source of information to understand people’s TV consumption (Mandryk & Inkpen, 2004) as it “represents a summary of the whole experience” elaborated “after exposure” (Sukalla, Bilandzic, Bolls, & Busselle, 2015), this still remains today's dominant approach.

Recently, a few researchers started to collect quantitative data such as log files of actions from streamers and Smart TVs, and browser history data (Bentley et al., 2019, Schweidel and Moe, 2016, Trouleau et al., 2016) to classify viewing behaviours. Despite their contributions, further limitations were highlighted. For example, relying on participants sharing the historic data with the researcher may lead to data manipulation (e.g., erasing of some data entries). Moreover, these approaches do not register interactive actions such as pause and rewind. BWDAT makes an effort to overcome the above mentioned limitations of current binge-watching studies and tools.

In fields such as cinema, the performing arts and video games, analyzing users’ experiences through the combination of both objective and subjective data has a noteworthy trajectory (Cox et al., 2020, Reeves et al., 1999). For instance, in controlled environments, Sukalla et al., 2015, Heiselberg and Bjorner, 2018 have combined both psychophysiological data (e.g., heart rate, skin conductance, facial EMG, eye tracking, electroencephalogram activity) and self-reported data to explore narrative engagement and audience's responses to Danish Broadcasting Corporation shows, respectively. Mixed methods approaches allow the researcher to validate self-reported data (Sukalla et al., 2015) and tackle known issues of under and over reporting information about, for instance, time spent online (Araujo et al., 2017, Scharkow, 2016) and phone usage (Boase & Ling, 2013). Moreover, such mixed approaches allow to take into consideration both the conscious and unconscious reactions of TV audiences (Heiselberg & Bjorner, 2018). Despite these benefits, there is a paucity of research on binge-watching that adopts this holistic approach, partially due to the lack of non-intrusive and open-access tools that facilitate it (Castro, Rigby, Cabral, & Nisi, 2019). BWDAT has the potential to help researchers to fill in this notorious gap.

In this paper, we argue that real-time collection of users’ data in natural environments and the combination of objective and subjective data can help researchers to delve deeper into the binge-watching phenomenon, and position BWDAT as an enabling tool in these regards. We also believe that the widespread use of wearable devices equipped with increasingly accurate sensors is opening up new opportunities to collect physiological data in the wild (e.g., measuring the autonomic nervous systems and stress levels) and match psychological traits to this data.

The aim of this article is to report on the design and evaluation of the technical implementation of the BWDAT^4^ tool. BWDAT is a non-intrusive and low-cost analytical research software that facilitates a holistic understanding of binge-watching in an uncontrolled environment remotely (e.g., the home). More specifically, the tool facilitates the collection of data about the pre-viewing, the viewing and the post-viewing of Netflix experiences, on viewers’ computers. This is achieved by combining, in a single tool, the collection of users' self-reported data with their physiological data, and their interactions with a VOD interface. With the design of this tool we aim to contribute to the existing debates about binge-watching and its multifaceted effects on people by allowing researchers to collect more thoroughly and efficiently data on VOD consumption, as well as tackling old and newly emerging research questions on the topic. BWDAT was designed, tested and successfully validated as a support tool for an investigation about excessive media use on Netflix, a research project carried out at University of Fribourg (Switzerland). Furthemore, BWDAT was created to facilitate longitudinal cross-national comparative studies on the topic of VOD consumption and on binge-watching, in particular.

## 2 Method

### 2.1 Design goals

The specific goals of BWDAT data-driven approach can be summarized as follows:

(a) The development of an application that records users’ interactions (e.g., pauses, forward) with VOD interfaces (currently supporting Netflix) and that assists in the synchronization of the physiological data with the content watched. This synchronization happens in such a way that the researcher can clearly pinpoint, for instance, the exact scene when a user performs a specific action (e.g., pauses the video).
(b) The development of a flexible smartwatch app that accurately collects physiological data (i.e., heart rate and inertial data) during VOD consumption at home. We opted for these two kinds of data because the heart rate reflects emotional activity (Mandryk & Inkpen, 2004), and wrist inertial data allows for a better interpretation of the heart rate due to the impact that movement has on the cardiovascular system. Furthermore, these physiological signals are the most popular and accurately registered by mainstream wearables.
(c) The design of a graphical interface that integrates and displays all the data collected by the different devices, to ensure its accurate interpretation. Furthermore, a set of data analysis reports were developed to integrate the BW sessions’ data, and help identify its errors.

### 2.2 Tools for data collection

BWDAT was integrated by a custom made browser extension, to function on Google Chrome.^5^ The version introduced here was designed for Netflix and collects up to eleven user actions on the interface during a viewing session^6^: open a Netflix tab, close a Netflix tab, content search, play, pause, forward, rewind, skip credits, skip intro, login and logout. Moreover, the browser extension allows for certain actions (e.g., closing Netflix) to trigger specific questionnaires created *ad hoc* by the researchers according to their research questions or hypothesis. To make sure that the participants filled the questionnaires after the session, and/or in case they accidentally closed the questionnaire before completing it, a pinned fix tab was added to the browser with all the questionnaires’ URLs, for later retrieval.

**Fig. 1:**
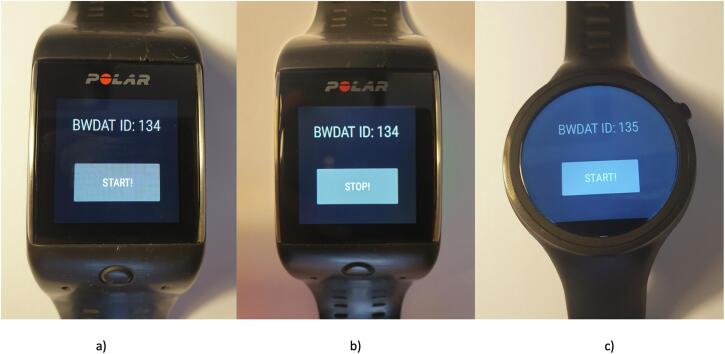
BWDAT smartwatch App a) Initial screen on Polar m600. b) Screen while collecting data. c) BWDAT in a round screen smartwatch moto 360.

**Fig. 2:**
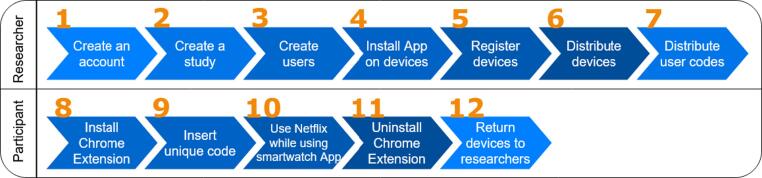
BWDAT usage diagram (grey - researcher, blue - participant). (For interpretation of the references to colour in this figure legend, the reader is referred to the web version of this article.)

**Fig. 3:**
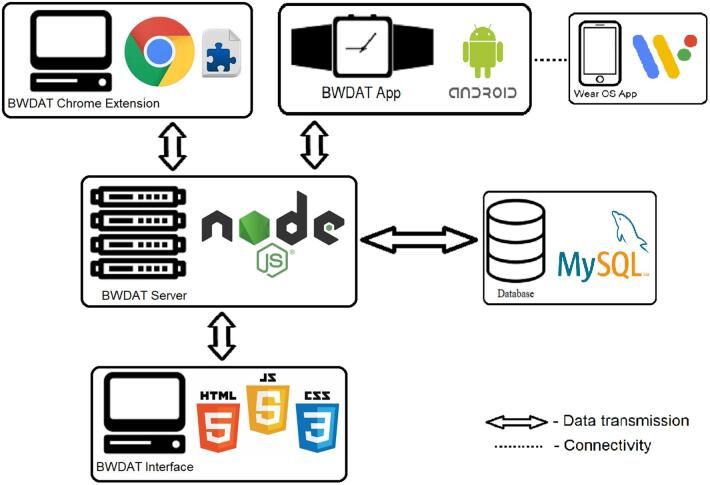
BWDAT data flow final architecture.

The App developed in Java, using Android Studio, collects heart rate (HR), gyroscope and accelerometer data. By using a high abstraction level approach, the App is currently supported by 26 different smartwatch models. The screen displays the smartwatch ID and a start and stop button (see Fig. 1) to initiate and finalize the physiological data collection process. The current values are sent to the server every second. Fig. 2 is a diagram of the different steps that need to be taken to use BWDAT. Fig. 3 represents the final architecture of BWDAT.

To develop and evaluate the technical quality of BWDAT, we used an iterative and incremental approach which involved several cycles of evaluations and validations. After a successful stress test, simulating the transmission of 20 smartwatches simultaneously to the database for 10 min, two trials and a long-term study were conducted. These three phases are described in detail in the next section.

### 2.3 Phases and participants

The first and second trial lasted one week. Participants’ involvement in these two trials was totally voluntary, and curiosity about the project was their main motivation to participate. Prior to the two 7-day trials, participants completed a pre-study questionnaire to collect demographic and viewing habits data and provided informed consents about their participation. Table 1 below synthesizes both trials specific goals, participants' requirements and profiles, and the stimulus used.

**Table 1:** Trials information synthesis.

Trial	Goal(s)	Participants’ profiles	Requirements	Main Stimulus^1^
#1	– To identify software installations and usability problems – To evaluate the tool for at least 2 h, the average binge-watching session identified by both Rigby, Brumby, Cox, and Gould (2018) and Castro et al. (2019) – To generate a larger scale data-set with real data to develop useful data report’s templates, and clean graphical data display	– 10 (8 males, 2 females) – 23–27 year-old (M = 25.7, SD = 1.3)	(a) to hold a Netflix account or be willing to open an account for the trial, (b) to posses a smartphone and a laptop with Google Chrome, (c) to be available and interested in watching a specific media content	*The Boy Who Harnessed the Wind,* new release in the moment the trial took place and it runs for almost two hours
#2	– To validate corrections MADE to the Chrome Extension BASED ON the errors identified IN trial 1	– 9 (all male) – 23 and 27 (M = 24.78, SD = 1.6)		*The Haunting of Hill House* (Ep. 1, Season 1), expected stronger HR variations, given the horror genre of the show

1 The participants were also free to watch any other content they liked.

Finally, after the refinements and iterations induced by the two trials findings, we considered the prototype ready for testing for its original purpose. BWDAT was hence used by a team of researchers for a long term study on binge-watching and its effects on sleep patterns. Our goal with that study was to evaluate the software performance on a larger scale, using more participants with a diverse demographic, and to compare its performance under 2 different environments (binge-watching and non-binge-watching mode). 37 participants (11 male and 26 female) between 18 and 52 years of age (M = 26.87, SD = 5.37) living in (Switzerland) were recruited. The research study divided participants into two groups (A and B). Participants in Group A (n = 21) were asked to watch three full episodes of a program of their own choice in a row. Participants in Group B (n = 16) were asked to only watch one episode in one sitting for three separate nights. All viewing sessions happened at home at night. The period of data collection lasted from June 2019 to September 2019 (4 months). The research team offered a 40 CHF compensation for participating in the study.

### 2.4 Procedure and devices used

Two days before trial 1 and 2, a researcher met the participants and explained the trials aims, presented the ethical issues and participants' rights, and provided each participant with a Polar m600 smartwatch.^7^ For trial 1, a participant used a Moto 360 smartwatch to confirm the flexibility of the App. For trial 2, the usage of the smartwatch was not mandatory, since the main aim was to verify the improvements to the browser extension. In all cases, participants followed the instructions displayed on the smartphone and smartwatch to connect and synchronize the devices. The use of their own smartphones did not raise any concerns among participants since it was clearly explained that the app would not invade or collect any of their other personal data. After receiving their unique user code on a private WhatsApp message, participants installed the browser extension following a URL shared through a WhatsApp group created to facilitate the communication between participants and the researcher during the trial. Once the browser extension was installed, a login page prompted the users asking them to insert their unique code.

In the long-term study, a total of nine Polar m600 smartwatches and nine mobile devices (5 Nokia Smartphones and 4 Samsung Tablets Galaxy Tab E) were used. Participants were provided with both a smartwatch and a smartphone that were already paired. This difference in regards to trial 1 and 2 is related to the ethical issues the project needs to meet (e.g., not to request the participants to use their personal Google accounts). Ethics approval for the study was granted by the Ethics Review Board of University of Fribourg.

All participants of the long-term study used their personal computers to watch content on Netflix. They received an email with their personal ID code, the URL to the browser Extension, and the URLs to the different questionnaires (pre- and post-study questionnaires, and the ones participants needed to complete before and after each session). To better understand the engagement of participants with each session’s content, the browser displayed a pre-session and post-session questionnaire containing, for instance, questions related to uses and gratifications and the PANAS scale (see Watson, Clark, & Tellegen, 1988).

### 2.5 Evaluation metrics

Interfaces and scripts were developed (using JavaScript, HTML and CSS), first, to identify the errors during the trials, and secondly, to provide the best interface for the data analysis and performance evaluation. The software offered an option to download data to be exported to other platforms (e.g., R Studio or Excel), allowing BWDAT to be used in other contexts and purposes. To frame our evaluation of the BWDAT tool, we distinguish between three working definitions of users’ sessions:

(a) Valid session: A session during which a participant consumes media content and which starts with “Session started” (opening a Netflix tab on the browser) and ends with “Session ended” (closing the Netflix tab).
(b) Semi-valid session: A session during which a participant consumes media content but the browser extension does not detect the “closing” of the Netflix tab.
(c) Empty session: A session during which the participant did not consume any type of media content, despite the session being bounded by the “Session started” and “Session ended” actions.

To evaluate the data collection accuracy, we took into consideration: (1) the coherence of the sequence of actions of the Netflix (e.g., some Play actions are followed by a Pause of the same episode) and (2) the accuracy of the concept of session implemented by the tool (i.e., that the participant manages to close the tab when the session is over). To evaluate the performance of the smartwatch app, we looked into the coverage and data transmission of each valid session and semi-valid session.

(a) Data Coverage (DC) defined as the % of the duration of the session for which physiological data was registered since it started until the end or the last action registered. For example, a 1 h session should have 3600 physiological data point values.
(b) Data Transmission (DT) defined as the percentage of physiological data points collected since the participant presses “Start” on the smartwatch until the end of the session.
(c) Content Coverage (CC) defined as the coverage of the media moment interactions while the content is played.
(d) Content Transmission (CT) is the same as CC, but it considers the start of the session when the participant presses “Start” on the smartwatch, validating the beginning of the session.

## 3 Results

### 3.1 Trial 1

During the seven-day trial period, the installation of both the smartwatch app and the browser extension ran smoothly. No problems were encountered or reported. The only remark is that some smartwatches took more time to update to Wear OS 2.0 due to the low battery levels of some of these devices at the moment the participant was installing the app. During the seven days, the researcher used the WhatsApp group to remind the participants about the importance of maintaining the devices charged.

**Fig. 4:**
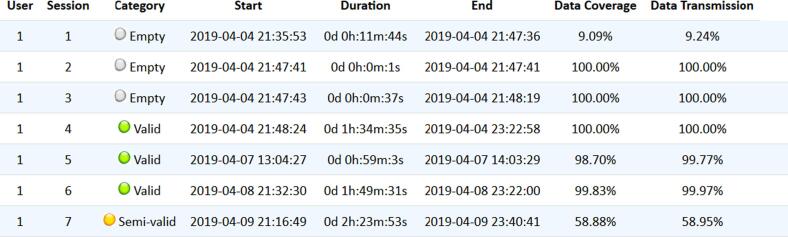
Sessions HR coverage data and classification interface (Output from BWDAT).

**Fig. 5:**
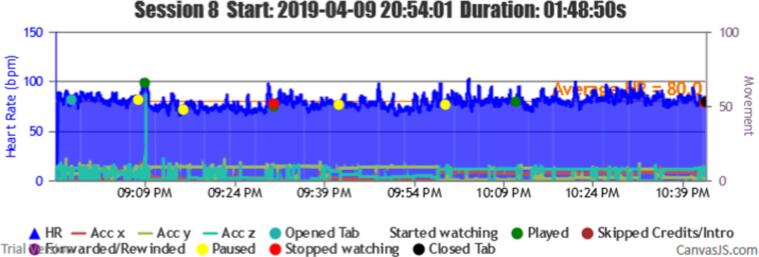
Session data representation (Output from BWDAT).

During the trial period, a total of 112 sessions were registered from the 10 participants. A graphical interface classified and provided the HR coverage of all the sessions, as shown in Fig. 4. Each session and its correlated information can be represented individually (see Fig. 5).

From all the sessions (see Table 2), 46.4% (n = 52) qualified as valid sessions, based on the definitions provided in Section 2.5. Data from the empty sessions reveals that between 1 and 11 (M = 5.4) empty sessions were registered in the very beginning of the study, when participants were logging in to the browser extension for the first time, or getting used to the Netflix platform (i.e., those who have never had a Netflix account before the trial). Therefore, the percentage of empty sessions registered is interpreted as a natural behaviour of users opening and closing the Netflix site, rather than the tool’s error.

**Table 2:** HR coverage of Trial 1.

Category	#	DC	DT	CC	CT
Valid Sessions	52	79.00%	80.65%	81.61%	83.48%
Valid Sessions with HR	47	88.00%	89.23%	90.29%	92.36%
Semi Valid Sessions	6	57.60%	61.05%	70.94%	71.04%
Semi Valid Sessions with HR	5	69.11%	73.26%	85.13%	85.24%

In 12.5% (n = 14) of the 112 sessions (6 of the 58 non-empty sessions [10.34%]), the action “closing Netflix tab” was not registered. At the end of the study we debriefed participants about these semi-valid sessions. Users explained that they did not close the Netflix tab immediately after the content was over.

Instead, they closed the tab a few hours later (e.g., after falling asleep) or they forgot that they were being monitored, leaving Netflix open and closing it days after, in places with no internet connection. Moreover, users described their experience with the tool as “natural” and “non-intrusive” of their viewing experiences.

**Fig. 6:**
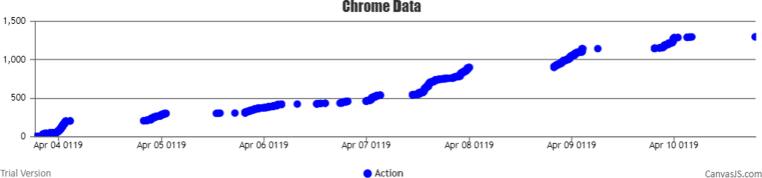
Graphical distribution in time of the total number of actions collected during Trial 1 (Output from BWDAT).

In total, from all sessions, 1,382 actions were registered along the week (see Fig. 6). The analytics interface facilitated the researchers in the analysis of the data and it allowed them to detect Netflix usability patterns. In 94.83% (n = 55) of the sessions, participants paused, rewind and/or forwarded the video, which highlights the importance of accurately registering these user actions to be able to effectively synchronize the content watched with the HR and inertial data.

Several issues emerged when some participants switched from one episode to another of the same program. For instance, in seven sessions with content, empty actions with no episode associated were registered when switching between episodes, meaning that the software was not fast enough to read the current content. Consequently, it mistakenly stamped the episode with the time frame of the previous one. In some cases, participants refreshed the Netflix page. In these cases, the first action registered by the browser extension was “empty content” followed by “Play” when the content was loaded. All the identified issues (e.g., actions with missing content and several empty sessions with no distinction between login and browsing) in trial 1 were fixed before trial 2 took place.

The 10 sessions of trial 1 containing the requested movie had an average of 89.10% of CC and an average of 89.17% CT. From those, 2 had a lower CC (25.78% and 65.93%) while all the other sessions had more than 99.3% CC.

In this first trial, the most extended session registered was 6 h long, as the participant fell asleep while binge-watching. The Polar m600 battery lasted long enough for the required and desired time stamps to be recorded. Also the participant with the moto m360 smartwatch had 100% data coverage of the content during the session, confirming the flexibility of the tool to serve on more than one smartwatch model.

### 3.2 Trial 2

Similarly to trial 1, all participants were briefed and filled in a pre-study questionnaire. No issues aroused while installing the tools. Because six participants installed the app a few days after receiving the smartwatch, the watch took more time to update to 2.0 OS due to the smartwatch battery levels being depleted after a few days of inactivity. Moreover, all participants installed the browser extension successfully. In sum, the participants acknowledge that the procedure was easy to follow.

In total, trial 2 recorded 247 actions. From the 26 sessions registered, 53.84% (n = 14) of these sessions registered watched content (movie, series or documentaries) and 38.46% (n = 10) were valid sessions.

The HR coverage (see Table 3) low performance was justified because participant 6 did not use the smartwatch when watching Netflix, but only the browser extension, in 7 out of the 9 sessions.

**Table 3:** HR coverage of Trial 2.

Category	#	DC	DT	CC	CT
Valid Sessions	10	35.23%	35.26%	42.25%	42.25%
Valid Sessions with HR	47	82.20%	82.26%	99.05%	99.05%
Semi Valid Sessions	4	57.60%	61.05%	70.94%	71.04%
Semi Valid Sessions with HR	1	100.00%	100.00%	100.00%	100.00%

Most participants reported to have had a stressful week and not clearly remembering the instructions when performing the viewing session. In those sessions when the browser extension did not register the closing Netflix tab action, we considered the end of the session as the last action (pause or stopped watching) performed by the participant. For instance, participant 7 had the most extended session, of 44 h, with a DC 1.42%, but a CC of 94.31%.

Despite the results in the DC, the browser extension improvements performed more accurately (compared to the previous version) when users were changing from one episode to the next one, as well as in the recording of the episode length. If we analyze the sessions with HR data collected, when the participants watched *The Haunting of Hill House* (Ep. 1, Season 1), the official stimulus of this trial, they had a 98.86% CC and DT. One of the users (participant number 2) did not record HR data while watching the episode.

Overall, this trial highlighted a fault in the design of the study, where the usage of the smartwatch was not declared mandatory, and it decreased the HR coverage evaluation results. This failure implied some loss of data. After this second trial, the browser extension was updated adding a “search” action which allows the researcher see what content the user browsers before engaging in the consumption of a show.

An alarm system was also implemented in the browser extension to verify if any physiological data was collected five seconds before the beginning of a Netflix session. If no data was transmitted, a blocking pop-up message asks the user to verify the smartwatch. Finally, the browser extension was adapted, with minor changes, to the Mozilla Firefox browser, proving its flexibility and scalability from browser to browser.

### 3.3 Long-term study

After the issues identified and refined through trial 1 and 2, we estimated we had reached a stable and complete version of the tool to make it available to the researchers (currently open source on Github). At this time (May 2019) a team from University of Fribourg was involved in conducting the long term study about the effects of binge-watching on night sleep. We took this opportunity to test the tool in a real in-the-wild study, performed by colleagues of another institution.

The study was designed as a A/B study, where two groups of binge-watchers performed different activities and their data was compared at the end of the study. Participants in Group A had to watch three episodes in a row at night, before going to sleep, while Group B watched one episode per night before going to sleep for 3 nights in a row. Researchers from University of Fribourg intended to compare the effect of binge versus non binge-watching on sleep patterns.

#### 3.3.1 Group A

The 21 participants performed a total of 95 viewing sessions during the study. Of these 95, 27 were valid, 32 semi-valid and 36 empty sessions. Given the focus of the study on night sleep, from those sessions, we only considered the sessions with various episodes watched on a night schedule, and with HR data, making a total of 21 valid sessions and 4 semi-valid sessions (n = 25) (see Table 4).

**Table 4:** HR coverage of long term study (Group A).

Category	#	DC	DT	CC	CT
Valid Sessions	21	77.27%	77.67%	89.36%	89.45%
Semi Valid Sessions	4	82.36%	87.41%	85.44%	86.96%

**Fig. 7:**
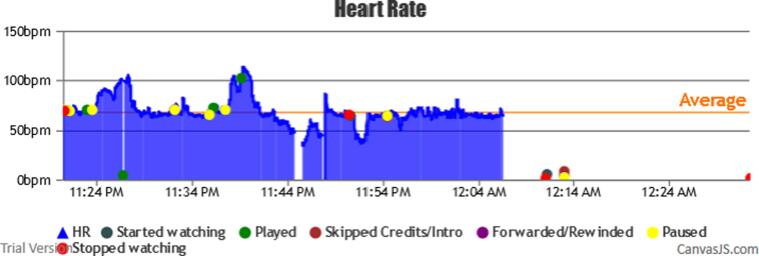
Smartwatch data failures (Output from BWDAT).

Several transmission failures, during data acquisition with the smartwatch and the server, impeded the system to record the information in the DB. The gaps of information lasted between 1 s to 2 min (M = 1 min and 13 s). All the sessions with less than 81% coverage stopped at a certain point in the session, which never recovered until the end, see Fig. 7 as an example. This way, some information was lost without the participants being aware of it. This issue was solved in the release of a new version (June 2020) by implementing an alarm system that verifies the data every 5 mins.

Some issues emerged in sessions with low physiological data coverage. Participant 9, which had a 62.55% DC, reported interruption and problems with the device. Participant 16, with a 34.38% DC, reported internet connection problems. Participant 18 answered the phone, which may have led to only an 80% DC, and participant 37 reported technical problems with the smartwatch battery, leading to a 90% DC and not 100%.

#### 3.3.2 Group B

Overall participants completed a total of 105 sessions during the study. Of the 105 sessions, 61 were valid, 3 semi-valid and 41 empty (see Table 5).

**Table 5:** HR coverage of long term study (Group B).

Category	#	DC	DT	CC	CT
Valid Sessions	61	76.66%	79.29%	87.25%	87.45
Semi Valid Sessions	3	57.60%	61.05%	70.94%	71.04%

**Fig. 8:**
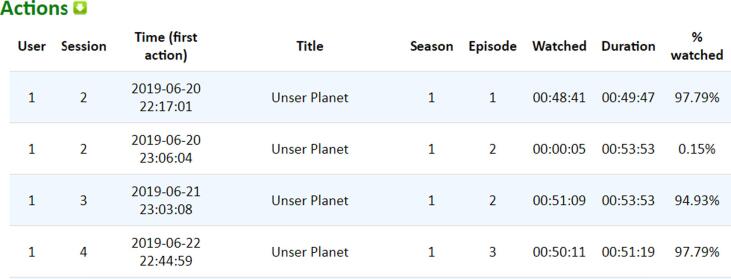
Content watched per session interface (Output from BWDAT).

**Fig. 9:**
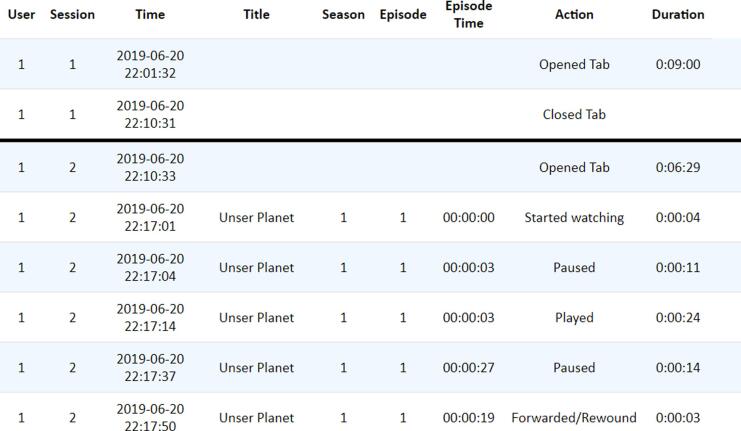
Actions recorded by session interface (Output from BWDAT).

**Fig. 10:**
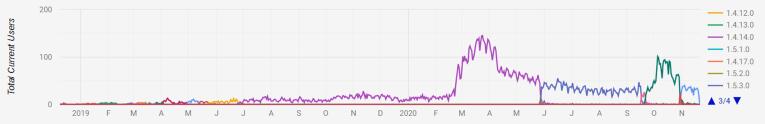
BWDAT weekly users per version since its first version (Output from Chrome Developers Interface on the 22nd November 2020).

Two interfaces (see Fig. 8 and Fig. 9) were implemented to analyze and download all the contents and actions of each session (see Fig. 10).

For group A and B alike, we considered as valid those sessions that include one or two watched episodes at night, before sleeping, with HR data and a post-session questionnaire associated (n = 51). The content watched had an average length of 42m23s, while the sessions lasted only 39m33s because of the skipping credits feature, and some cases of multiple forwarding while watching. Each night, the percentage of data coverage increased, possibly showing the users getting better at using the technology.

Regarding the browser extension, the only challenge was making sure that participants close the Netflix tab right after finalizing the viewing session. However, in those cases that they forgot to do so, we consider the last pause or stop playing action registered in that session as the end of the session. This approach worked well. In total, we registered 2,755 actions. A script was then developed to easily find errors in the data collected. For example, when the server received several actions at the same time this derived in some actions being registered in the wrong order.

After this long-term study the same version of BWDAT (V1.4.14, purple in Fig. 10), was used by the same research group at University of Fribourg for further studies. Data was collected from more than 200 users (n = 203) during one week, one order of magnitude above the previous study (Cabral et al., 2020). Finally, we debriefed the University of Fribourg researchers’ experience with the tool, and we received positive feedback. According to the researchers reports, these further studies run smoothly and the tool performed well without requiring any upgrades or changes in the software. The researchers also confirmed that the right data was captured to explore their hypothesis about binge-watching.

The main positive features highlighted by them were the automatic reports generated by BWDAT, which allowed the data analysis of thousands of data entries in a simplified way (Fig. 4, Fig. 8), and the clear data visualization (Fig. 5, Fig. 6). Researchers also praised BWDAT accuracy and flexibility with several languages and time zones (e. g. collecting Netflix actions of a Korean movie being watched in the USA (e.g., action “Clicked: ”)).

From evaluation results and the extensive feedback received, the first author refined the browser extension and in September 2020 a new BWDAT Chrome Extension version was released (V 1.6.2). New features included a *smartwatch's low battery alert* and *a next episode form display option* (if pre-defined, when the viewer moves to a new episode a form is displayed in a new tab). To easily identify to which viewing session each questionnaire belongs to, the session ID was added to the questionnaire URL.

Table 6 summarizes the main issues identified in the iterative design of the tool and the status of those issues.

**Table 6:** Identified errors in the software.

Phase	Error(s) identified	Status
Trial 1	– Empty content actions (Pause and Play) due to delays on reading episode’s info – The browser extension would read the time from the previous action when moving from one episode to another	Solved before starting trial 2
Trial 2	– Participants would forget to press ‘Start!” on the smartwatch before starting each session	Solved before starting long-term study
Long-term study	– Smartwatch would stop sending HR data – Participants wouldn’t notice low battery levels of the smartwatch	Solved for future studies

## 4 Discussion

Inspired by the benefits described by authors such as Heiselberg and Bjorner (2018) about the combination of objective and subjective data to analyze viewers’ behaviours, we created BWDAT. This paper reports on the design and technical evaluation framework of BWDAT, a tool created to facilitate the study of the viewing experience on VOD platforms − more precisely, on Netflix − in an uncontrolled environment and for long periods of time. To the best of our knowledge, this is the first open-access tool designed to collect both subjective and objective data for research on the use and consumption of VOD services

According to our tool evaluation results and researchers feedback, BWDAT provides researchers with a reliable instrument able to accurately synchronize users' interactions on Netflix with, on the one side, the physiological data generated by the user (and collected through a smartwatch) and, on the other side, synchronising such data with the timeline of the content watched. All of this with a minimal level of intrusiveness and supervision, and positive user experience.

Here we have described the iterative and incremental development and testing of BWDAT, explaining the main design decisions and technical obstacles and providing a framework to evaluate the reliability of these tools. BWDAT has already been used successfully in long-term (several months) research studies geared towards the understanding of different facets of Netflix content consumption. The tool also provides an analytics interface for the real-time data and automatically sends usage data to a back end server, which can be used for exporting the data to statistical packages to perform further analytics. Moreover, during the testing of BWDAT we designed an evaluation framework that provides a baseline for testing VOD experiences in-the-wild research tools.

The tool was tested over long periods and reported robust, high performance and availability (uptime) during sessions where participants spend several hours watching one specific unit of content (e.g., a movie, a documentary) as well as when binge-watching TV series. For instance, watching the movie requested on trial 1 obtained 89.10% coverage, and for the series episode on trial 2, 98.86% of content coverage and data transmission. Furthermore, the current version supports studies with more than 200 weekly users, proving its reliability and the success of the approach taken. These are positive and promising results regarding the robustness and reliability of the tool.

BWDAT was useful in exposing high-level user behavior that is essential for understading the binge-watching mode of viewing (e.g., viewers’ actions on Netflix interface, and the synchronization of the content watched with the viewers’ physiological data). The results from our trials demonstrate how the BWDAT tool and our framework can be easily used in a wide range of media research projects conducted not only in the wild, as the long-term study has proved, but also in a controlled environment. In the later scenario, the tool is flexible enough to incorporate data from sensors that measure, for instance, viewer’s temperature or electroencephalogram activity. Concerning the analytics interface, the synchronization of all the objective data in a single interface facilitates its interpretation by the research teams (e.g., what scenes increased viewer’s HR?). Similarly, the interfaces for action and data coverage analysis show that providing all the different data in the same platform enables automation scripts to analyze thousands of data entries in a matter of seconds.

### 4.1 Future work and limitations

Hitherto, BWDAT has been used by various teams of researchers to investigate various issues connected to binge-watching, such as to explore if binge-watching leads to a stronger immersion into the narration, and if binge-watchers develop further parasocial relationships than appointment viewers. However, BWDAT can also help explore how a user's interaction with the Netflix interface during a viewing session relates to self-reported levels of transportation (see Green & Brock, 2000). It can also be used to measure how the environment (controlled versus uncontrolled) of a media-related study affects viewing behaviours, or to explore if viewers’ interaction with an interface changes according to the content watched (e.g., in terms of genre and format of the media product). Although we applied BWDAT in the context of binge-watching at home, the intentional flexibility of its design guarantees its use in the more general domain of the consumption of streaming content in any type of environment where a laptop and Internet connection are both present (e.g., at work, whilst commuting medium/long distances in public transportation).

Despite the novelty of the BWDAT tool, we have identified several limitations that suggest paths for future work. First, a backup system on both the smartwatch and the browser extension should be developed to ensure that data is still gathered when the Wi-Fi connection is lost. Second, the technology used does not offer data about the potential multitasking behaviour of the viewer. Despite its intrusiveness, the usage of cameras (Rigby, Brumby, Gould, & Cox, 2017) could be a potential solution. Third, detecting if the users fall asleep (from the HR levels or body stillness) could help classify users’ behaviors. Fourth, considering the predominance of the TV set as the main screen to consume TV content at home, BWDAT should be adapted for smart TVs. The same applies to the consumption of VOD content on smartphones. Finally, the tool could be upgraded to register other types of physiological data (e.g., skin temperature) directly collected by newer generations of wearables or by uploading data from those devices into the analytical tool using .xlsx (Excel) or .csv files.

## 5 Conclusion

This paper presents BWDAT, a novel, reliable and flexible tool for the study of VOD consumption at home. We argue that the combination of subjective data and objective data collected in a non-intrusive fashion has the potential to challenge the existing knowledge regarding the effects of binge-watching. In this article, we described the design and technical evaluation of the tool to conclude that BWDAT works with standard devices and software, and provides researchers with an easy to use and deploy platform to holistically study the behaviour patterns of consumers of VOD media content.

We have explored the technical challenges and limitations of developing tools for in-the-wild studies and provided an evaluation framework for VOD content media consumption in general.

The combination of physiological data with user interaction and content description provides a powerful mechanism for research teams working at the intersection of this new interdisciplinary area and allowing for a more comprehensive and complete understanding of not only binge-watching behaviours but VOD viewing habits and experiences in general.

## CRediT authorship contribution statement

**José A. Cordeiro:** Software, Data curation, Investigation, Visualization, Writing - original draft. **Deborah Castro:** Conceptualization, Supervision, Writing - original draft, Writing - review & editing. **Valentina Nisi:** Conceptualization, Writing - review & editing. **Nuno J. Nunes:** Conceptualization, Supervision, Writing - review & editing.

## Declaration of Competing Interest

The authors declare that they have no known competing financial interests or personal relationships that could have appeared to influence the work reported in this paper.

## Footnotes

1 The term binge-watching co-exists with the more comprehensive and stigma-free concept of media-marathoning (Perks, 2015:ix) that emphasizes viewers’ engagement with a narrative world and viewers’ agency to decide upon the duration of a narrative journey.

2 See: https://www.statista.com/statistics/1107559/video-streaming-consumption-growth-worldwide-coronavirus/.

3 To have an overview of what previous researchers have done, we used the literature review conducted by Merikivi, Bragge, Scornavacca, and Verhagen (2019) as a starting point, which we updated with the academic literature published until May 2020.

4 BWDAT follows the aim of the B-Watch tool, described by (Cabral et al., 2020) but with a new sustainable and dynamic approach. The open code, the open access URL to the tool and the manual to use it, are all available in the following link: https://github.com/JoseDAssisCordeiro/BWDAT.

5 The most commonly used browser in the web (63.8%): https://www.w3counter.com/globalstats.php, visited on the 23rd November 2020.

6 A session is defined as the time between the opening of a Netflix tab and its closure.

7 We evaluated several devices combining reliability, cost, ease of development and autonomy, and compatibility with Wear OS 2.0 (https://wearos.google.com) operating system, which allows for standalone applications. The Polar m600 smartwatch Android-based wearable was selected. A technical test was run with Polar m600, by comparing the data collected from 4 users with Empatica E4, giving an average mean absolute error of 5.31%. According to Nelson and Allen (2019), an average absolute error below 10% serves as an indicator of an acceptable heart rate accuracy.

## References

[b0005] Araujo T., Wonneberger A., Neijens P., de Vreese C. (2017). How much time do you spend online? Understanding and improving the accuracy of self-reported measures of internet use. Communication Methods and Measures..

[b0010] Basterra-Gortari F., Bes-Rastrollo M., Gea A., Nunez-Cordoba J., Toledo E., Martinez-Gonzalez M. (2014). Television viewing, computer use, time driving and all-cause mortality: The sun cohort. Journal of the American Heart Association.

[b0015] Bentley, F., Silverman, M., & Bica, M. (2019). Exploring online video watching behaviors. Proceedings of ACM International Conference of Interactive Experiences for TV and Online Video (TVX 2019), Manchester, UK ACM, New York, USA. https://doi.org/10.1145/3317697.3323355.

[b0020] Berger A.A. (1978). The hidden compulsion in television. Journal of the University Film Association.

[b0025] Boase J., Ling R. (2013). Measuring mobile phone use: Self-report versus log data. Journal of Computer Mediated Communication.

[b9010] Cabral D., Castro D., Rigby J.M., Vasanth H., Cameirão M.S., Bermúdez i Badia S., Nisi V., Nunes N.J., Ma L., Wang M., Correia N., Paz Z. (2020). To binge or not to binge: Viewers' moods and mehaviours during the consumption of subscribed video streaming. Entertainment Computing - ICEC 2020. Lecture Notes in Computer Science.

[b9000] Castro D., Rigby J.M., Cabral D., Nisi V. (2019). The binge-watcher's journey: Investigating motivations, contexts, and affective states surrounding Netflix viewing. Convergence: The International Journal of Research into New Media Technologies.

[b0030] Cox, A., Bianchi-Berthouze, N., & Jennett, C. (2020). The use of eyetracking for measuring immersion. CogSci 2006 Workshop: What have eye movements told us so far, and what is next.

[b0035] Exelmansa L., Van den Bulckb J. (2017). Binge viewing, sleep, and the role of pre-sleep arousal. Journal of Clinical Sleep Medicine: JCSM : Official Publication of the American Academy of Sleep Medicine.

[b0040] Flayelle M., Maurage P., Billieux J. (2017). Toward a qualitative understanding of binge-watching behaviors: A focus group approach. Journal of Behavioral Addictions.

[b0045] Flayelle M., Maurage P., Di Lorenzo K.R., Vögele C., Gainsbury S., Billieux J. (2020). Binge-watching: What do we know so far? A first systematic review of the evidence. Current Addiction Reports.

[b0050] Green M., Brock T. (2000). The role of transportation in the persuasiveness of public narrative. Journal of Personality and Social Psychology.

[b0055] Heiselberg L., Bjorner T. (2018). How to evaluate emotional experiences in television drama series: Improving viewer evaluations by psychophysiological measurements and self-reports. Behaviour & Information Technology.

[b0060] Jenner M. (2015). Binge-watching: Video-on-demand, quality tv and mainstreaming fandom. International Journal of Cultural Studies.

[b0065] Mandryk, R., & Inkpen, K. (2004). Physiological indicators for the evaluation of co-located collaborative play. Proceedings of the ACM Conference on Computer Supported Cooperative Work, CSCW, 102-111. https://doi.org/10.1145/1031607.1031625.

[b0070] Merikivi J., Bragge J., Scornavacca E., Verhagen T. (2019). Binge-watching serialized video content: A transdisciplinary review. Television & New Media.

[b0075] Nelson, B., & Allen, N. (2019). Accuracy of consumer wearable heart rate measurement during an ecologically valid 24-hour period: Intraindividual validation study. Study JMIR Mhealth Uhealth 2019, 7(3):e10828. https://doi.org/10.2196/10828. 10.2196/10828 PMC6431828 30855232

[b0080] Panda S., Pandey S. (2017). Binge watching and college students: Motivations and outcomes. Young Consumers.

[b0085] Perks L.G. (2015). Media Marathoning: Immersions in Morality.

[b0090] Pierce-Grove R. (2016). Just one more: How journalists frame binge watching. First Monday.

[b0095] Reeves B., Lang A., Kim E., Tatar D. (1999). The effects of screen size and message content on attention and arousal. Media Psychology.

[b0100] Riddle K., Peebles A., Davis C., Xu F., Schroeder E. (2017). The addictive potential of television binge watching: Comparing intentional and unintentional binges. Psychology of Popular Media Culture.

[b0105] Rigby, J., Brumby, D., Gould, S., & Cox, A. (2017). Media multitasking at home: A video observation study of concurrent tv and mobile device usage. In proceedings of the 2017 ACM International Conference of Interactive Computing Machinery, New York, NY, USA, 3-10. https://doi.org/10.1145/3077548.3077560.

[b0110] Rigby, J., Brumby, D., Cox, A., & Gould, S. (2018). Old habits die hard: A diary study of on-demand video viewing. In CHI EA '18: Extended Abstracts of the 2018 CHI Conference on Human Factors in Computing Systems, Association for Computer Machinery, New York, NY, USA, Paper LEW016, 1-6. https://doi.org/10.1145/3170427.3188665.

[b0115] Scharkow M. (2016). The accuracy of self-reported internet use - A validation study using client log data. Communication Methods and Measures.

[b0120] Schweidel D., Moe W. (2016). Binge watching and advertising. Journal of Marketing.

[b0125] Shirakawa T., Iso H., Yamagishi K., Yatsuya H., Tanabe N., Ikehara S., Tamakoshi A. (2016). Watching television and risk of mortality from pulmonary embolism among japanese men and women. Circulation.

[b0130] Starosta J., Izydorczyk B., Lizinczyk S. (2019). Characteristics of people’s binge-watching behavior in the “entering into early adulthood” period of life. Health Psychology Report.

[b0135] Steiner E., Xu K. (2018). Binge-watching motivates change: Uses and gratifications of streaming video viewers challenge traditional tv research. Convergence: The International Journal of Research into New Media Technologies.

[b0140] Sukalla F., Bilandzic H., Bolls P., Busselle R. (2015). Embodiment of narrative engagement connecting self-reported narrative engagement to psychophysiological measures. Journal of Media Psychology Theories Methods and Applications.

[b0145] Trouleau, W., Ashkan, A., Ding, W., & Eriksson, B. (2016). Just one more: Modeling binge watching behavior. KDD ’16: Proceedings of the 22nd ACM SIGKDD International Conference on Knowledge Discovery and Data Mining, 1215-1224. https://doi.org/10.1145/2939672.2939792.

[b0150] Van den Bulck J. (2000). Is television bad for your health? behavior and body image of the adolescent “couch potato”. Journal of Youth and Adolescence.

[b0155] Watson D., Clark L.A., Tellegen A. (1988). Development and validation of brief measures of positive and negative affect: The PANAS scales. Journal of Personality and Social Psychology.

