# Supplementary material for: BWDAT: A research tool for analyzing the consumption of VOD content at home
Source: Addict Behav Rep. 2020 Dec 30;13:100336. doi: 10.1016/j.abrep.2020.100336 (PMC7889796; doi:10.1016/j.abrep.2020.100336)
Supplement: Supplementary data 1 [file mmc1.pdf]

Binge-Watching Data Analysis Tool

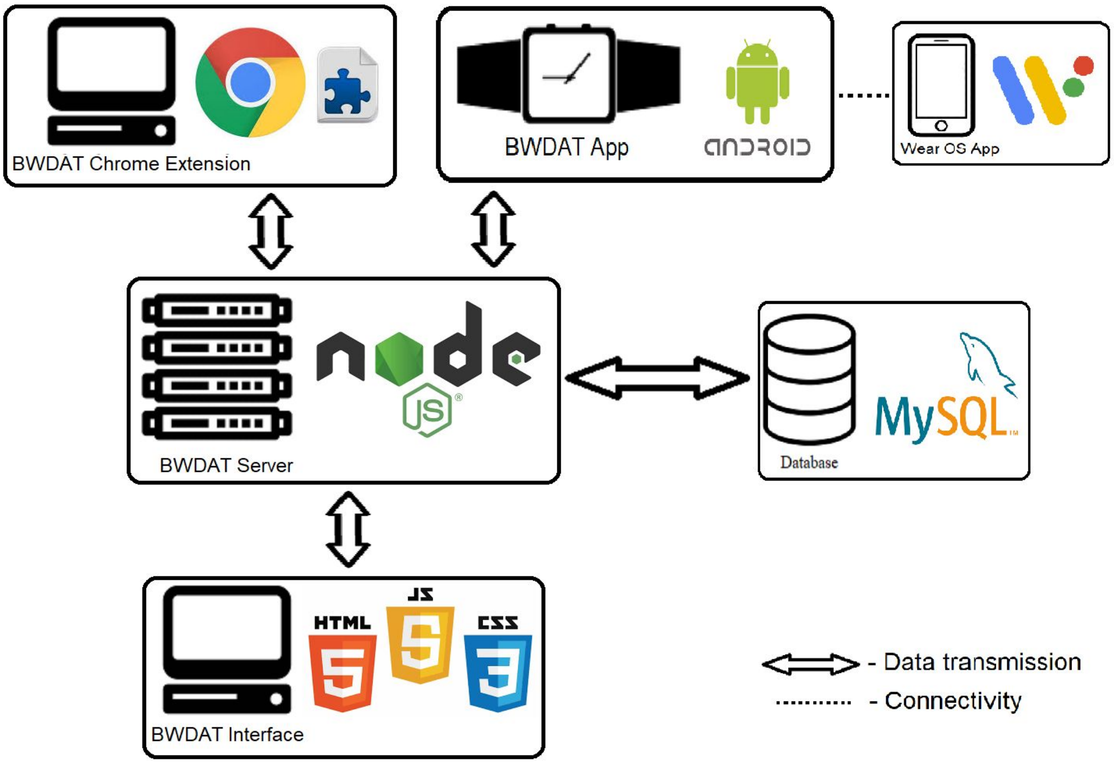

BWDAT Manual

José d'Assis Cordeiro

TÉCNICO LISBOA

Overview

The aim of this tool is to gather and analyse users' interactions with Netflix interface and participant's heart rate and wrist movements during each viewing session.

The tool is divided in 5 parts:

- Chrome Extension
- Smartwatch Application
- Server
- Database
- Admin Interface

The Netflix interface actions are detected by the Chrome Extension. That component also allows the users to answer predefined forms before and after the study, in the beginning and end of each session. This allows them to report other relevant information for the study, if needed.

This information will be sent to a server to be stored in a database. By gathering all the data in a single place it is easier to handle and analyse it.

A web interface is provided to allow admins to create and edit new projects, manage the different users and devices associated with their projects.

It is possible to have different studies running at the same time, using several devices per user and download the data collected for each study.

Concepts: study, user and devices

A study is a project with a certain duration that uses one or more of the tools available on BWDAT. Currently it has a Chrome Extension (from now on Extension), on Chrome Web Store, a Mozilla Firefox Add-on (from now on Add-on), and an Application for Smartwatches (from now on Smartwatch App), on Google Play Store. The number of participants doesn't need to be defined in advance.

The users, when registering, have an unique string code attributed to them. They can use that code to login to activate the Extension or Add-on, once installed.

When the study requires the collection of physiological data, the devices need to be manually added to the corresponding study, once the App is installed on the device. The admin of the project has to record when a participant receives and returns them.

Chrome Extension

The BWDAT Chrome Extension was developed with JavaScript and the Google Chrome libraries, to collect all the user interactions with the Netflix' streamer and tabs, and to allow self-reported data forms. It is available on link.

How it works (the diagram starts on the green rectangle, and blue ones represent the participant's actions, and the yellow ones the software actions):

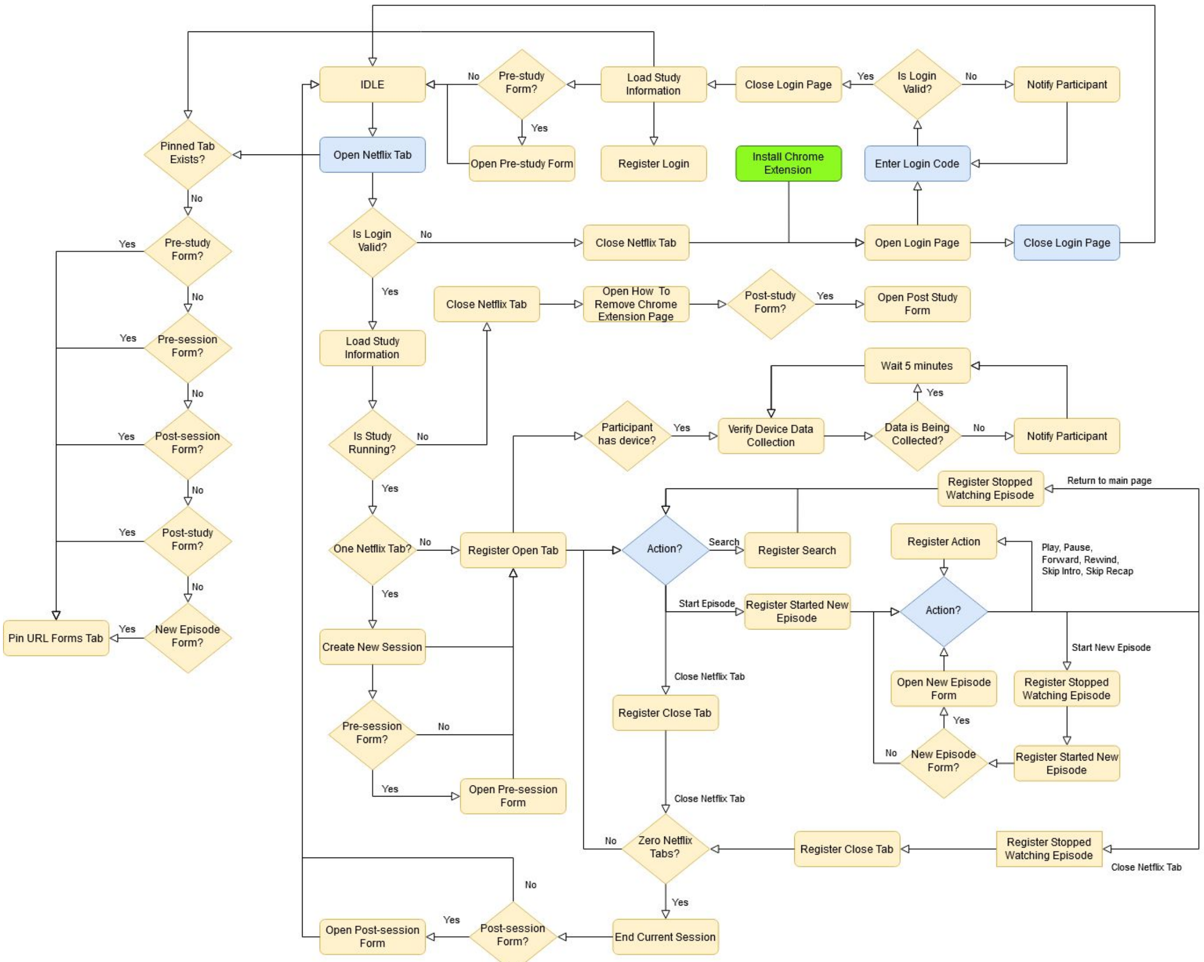

Diagram 1. - Chrome Extension

1st - The user installs the Chrome Extension. When attempting to open Netflix, a Login page will be forced until a valid Login code is inserted. When the code is wrong, a notification will be displayed. The time of the first Login is registered in the database.

2nd - Once the user is logged in, the information of the project is loaded into the Google Chrome Extension local memory. If the study has a pre-study form, that form is prompted in a new tab. If the project has a pre-study, pre-session, post-session, post-study or new epsode form, a pinned tab is added to the window with all the forms of the study as backup, and an email address is provided for direct help.

3rd - When the user opens a Netflix browser, the background script updates the project's information. If the project already ended, a "How to remove the Chrome Extension" page will be displayed and the Netflix tab is closed.

Otherwise, in case the study requires a pre-session questionnaire it will be opened in a new tab on the window. The beginning of a new session will be registered in the database. If the user has a device associated, the software verifies if physiological data is being collected. If not, the user will be warned.

4th - After replying to the pre-study form, the user starts interacting with Netflix's interface. The actions on the interface will be registered by myscript script and sent to the background script, that will manage and filter that information.

5h - During the session, a script listens to the events from the video streamer. The title, season, episode number and episode code will be read from the html code every 50ns. We consider it a movie or a documentary when there is no season and episode number information.

6th - When the user closes all the Netflix tabs, and if the project has a post-study questionnaire, the form is displayed. The time of closing tab is registered in the database.

Action	Description
Opened Netflix Tab	Participant opens a Netflix tab
Searched	Participant searches for a content in the search bar
Started watching	Participant starts to watch a certain episode
Played	Participant plays streaming content from a pause state
Paused	Participant pauses streaming content from a play state
Forwarded/Rewound	Participant jumps on the episode
Stopped watching	Participant stops watching a certain episode
Clicked: Skipped Intro	Participants skips the intro of the episode
Clicked: Skipped Recap	Participant skips the recap of the previous episode
Closed Netflix Tab	Participant closed a Netflix tab

Table 1. - Actions registered by Chrome Extension

To remove the BWDAT Extension go to chrome://extensions/, search for the BWDAT Extension, and click 'Remove'.

Smartwatch Application

To collect participant's heart rate and movements during each Netflix session, an Application for smartwatches was developed using Java and Google Android libraries. It is required that the smartwatch has a Wear 2.0 or higher OS, since it is a standalone App. The http requests are done directly by wi-fi to the router with no need for sending it through the smartphone. It is available on link. The smartwatch battery using this application lasts from 5h30 to 6h when fully charged.

How to install:

1st - Install Wear OS App on the smartphone. Turn on the Bluetooth on both smartwatch and smartphone.

2nd - On the smartwatch send a synchronization request. Follow the steps on the phone. Make sure you synchronize the accounts.

3rd - After the synchronization, on the smartwatch, press the left button and click on Play Store App. Search for BWDAT using the provided screen keyboard and click install.

5th - Turn off bluetooth connection on Settings -> Connectivity -> Bluetooth and swipe left. (If the participant forgets this step, the App will turn off the Bluetooth once it starts collecting data.)

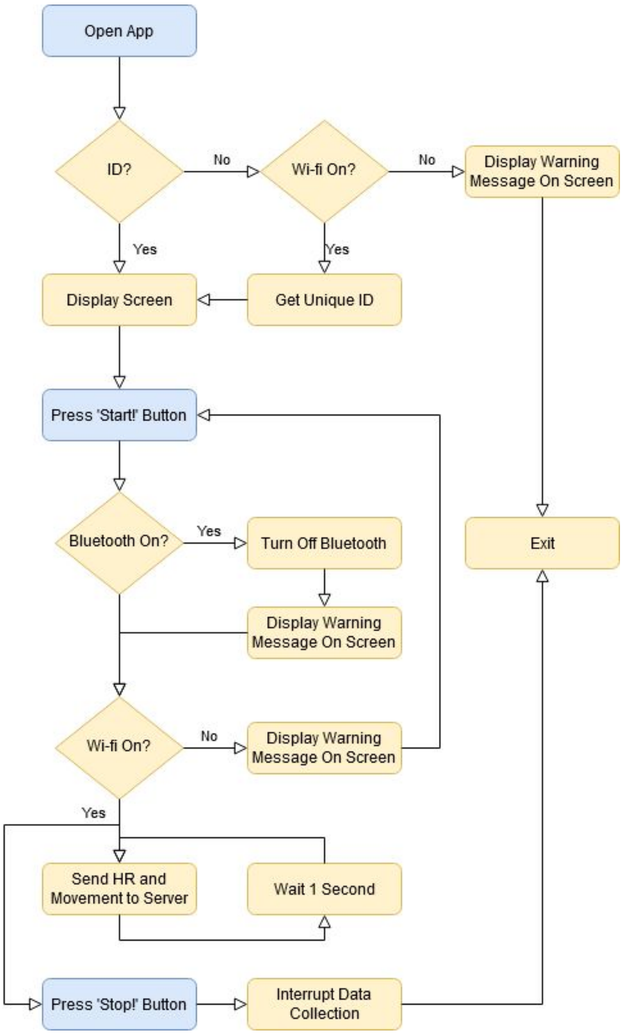

Diagram 2. - Smartwatch App

How it works:

1st - When the user opens the BWDAT App, if the local memory doesn't have a BWDAT ID registered, a new ID request is sent to the server and it is registered locally in the smartwatch App identifying that smartwatch. If there is no wi-fi connection, a warning is prompted on the screen.

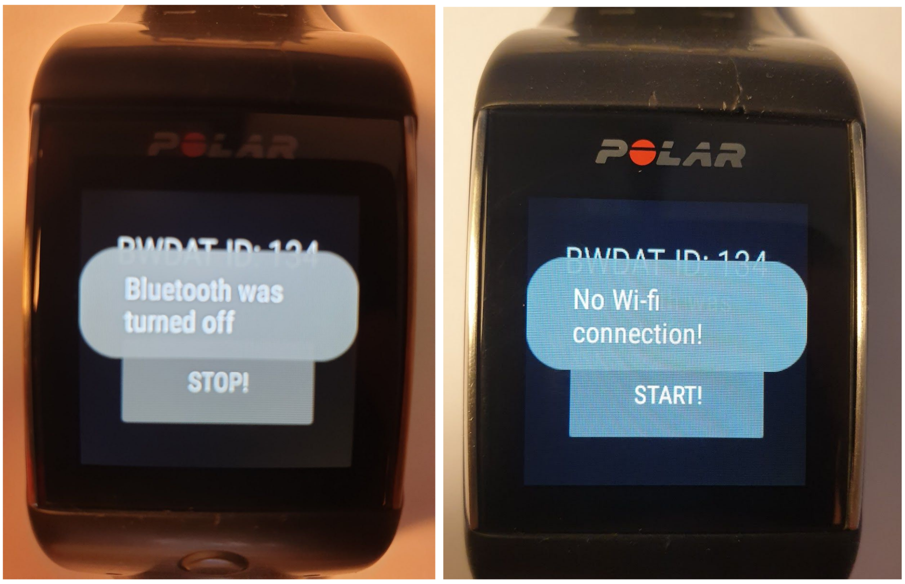

a) b)

Figure 1. - a) Bluetooth being turned off by the BWDAT App when the Start button is pressed. b) No wi-fi connection found user notification.

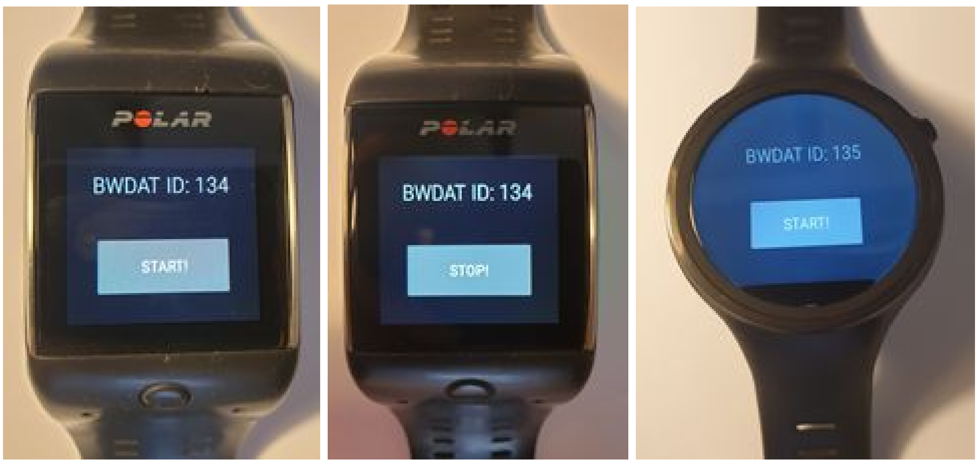

a) b) c)

Figure 2: BWDAT smartwatch App a) Initial screen on Polar m600. b) Screen while collecting data. c) BWDAT in a round screen smartwatch moto 360.

2nd - An initial screen displays a 'Start!' button to start a new session and that smartwach's ID. When pressed, the wi-fi connection is verified. After pressing 'Start!', the App updates the Heart Rate, Gyroscope and Accelerometer data every time it changes and sends it and the battery level every second to the server.

3rd - During the session the App is kept alive and sends https requests with the information using a periodic timer and a Wake lock, not allowing the smartwatch to go into ambient/sleep mode.

4th - When the participant presses the “Stop!” button in the interface, the information is no longer updated or sent to the server, and the application is closed.

Supported devices:
Asus ZenWatch, Fossil Sport, Fossil Wear, Huawei Watch 2, Huawei WATCH, LGE G Watch R, LG Watch Urbane 2nd Edition, LG Watch Urbane, LG Watch Sport, Mobvoi TicWatch C2, Tic Watch BT, TicWatch Pro, TicWatch Pro 4G, TicWatch C2, Ticwatch S/E Smartwatch, Montblanc SUMMIT, Motorola Moto 360 (2nd Gen), Motorola Moto 360, New Balance Run IQ, Polar M600 and Samsung Gear Live, Movado Connect 2.0, Suunto 7, TAG Heuer Connected 2020, Casio WSD-F21HR.

How to remove BWDAT App:
Settings -> Apps -> BWDAT -> Uninstall

Server

The BWDAT server was developed in Express JS (Node JS framework), and it deals with different requests made by the various components of the tool to the database. An open-access version is running on bwdat.rnl.tecnico.ulisboa.pt using the communication port 430 (https).

The requests are formatted in a JSON structure to have an header with the request, and the body the the information provided. The body input is verified to keep the application reliability. In order to maintain availability all the errors are written in an error log file.

The connection to the database is done using a pool of connections.

Database

The database was implemented in MySQL.
It was designed in order to allow new features to be added in the future.
The database schematic can be seen in Figure 3:

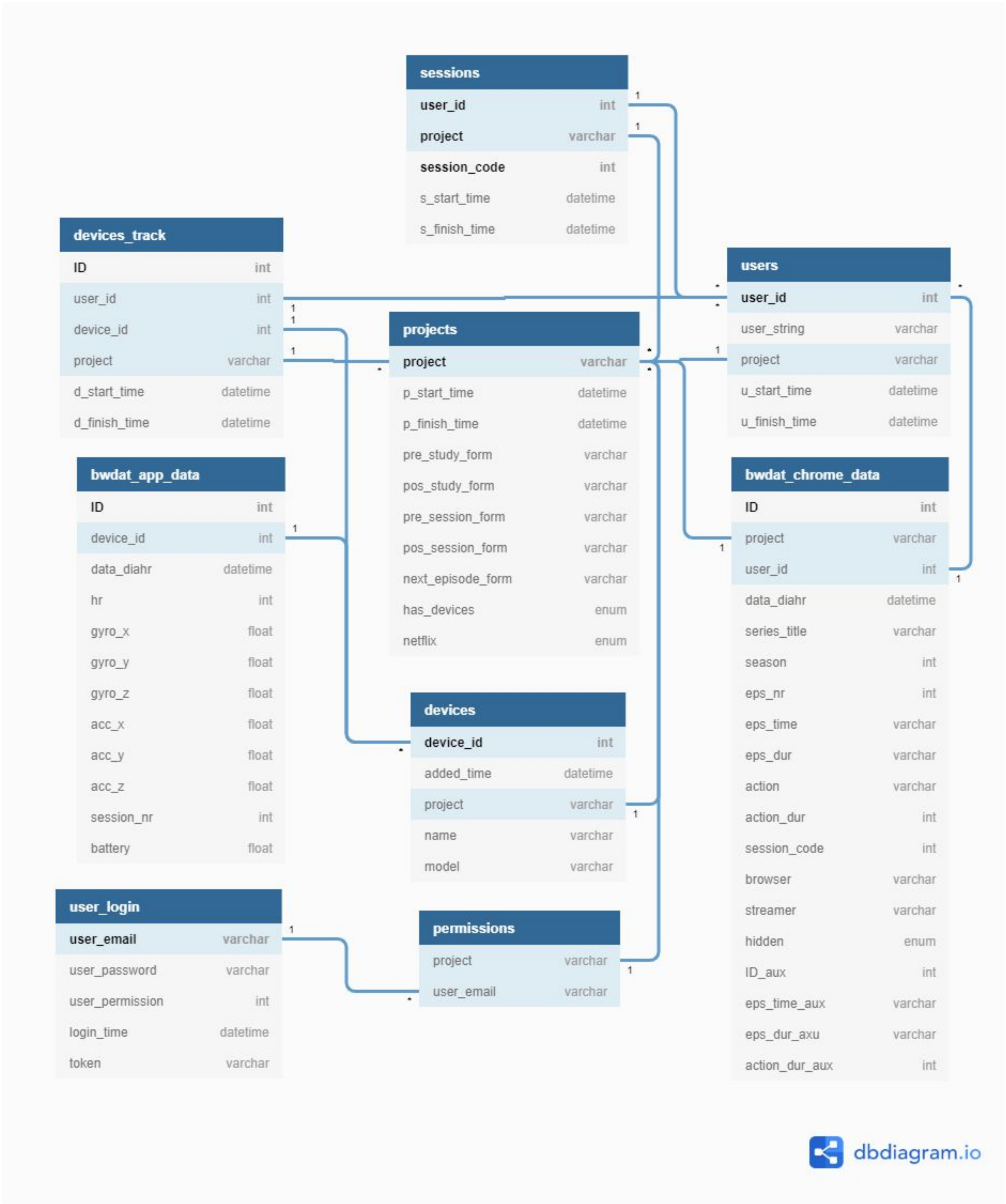

Figure 3. - Database representation

Admin Interface

An interface is used to manage all the data of the projects. It is possible to create a new project, edit an existing one, register new users for each project or user tests. It is also possible to assign devices to projects and keep track who is currently using which device and which devices are available. A login system protects invasive and malicious actions from other people.

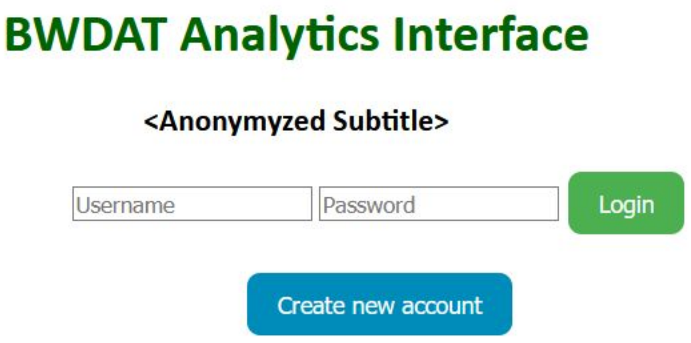

Figure 4. - BWDAT login interface

A list of the projects of that user then displayed on the main interface (Figure 5). There, the researcher can change the project's definitions (Edit > Project), add and remove users (Figure 6) (Edit > Users), and change its permissions (Edit > Permissions) by adding other admins to the study. In the user's interface it is possible to download each user's sessions excel file, or all the users in a .zip file.

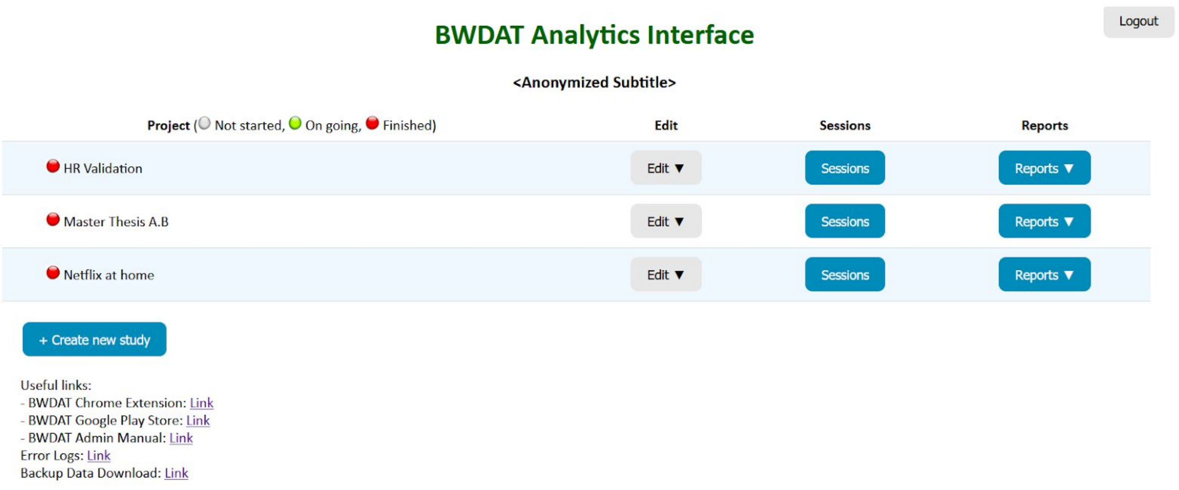

Figure 5. - Example of BWDAT main interface

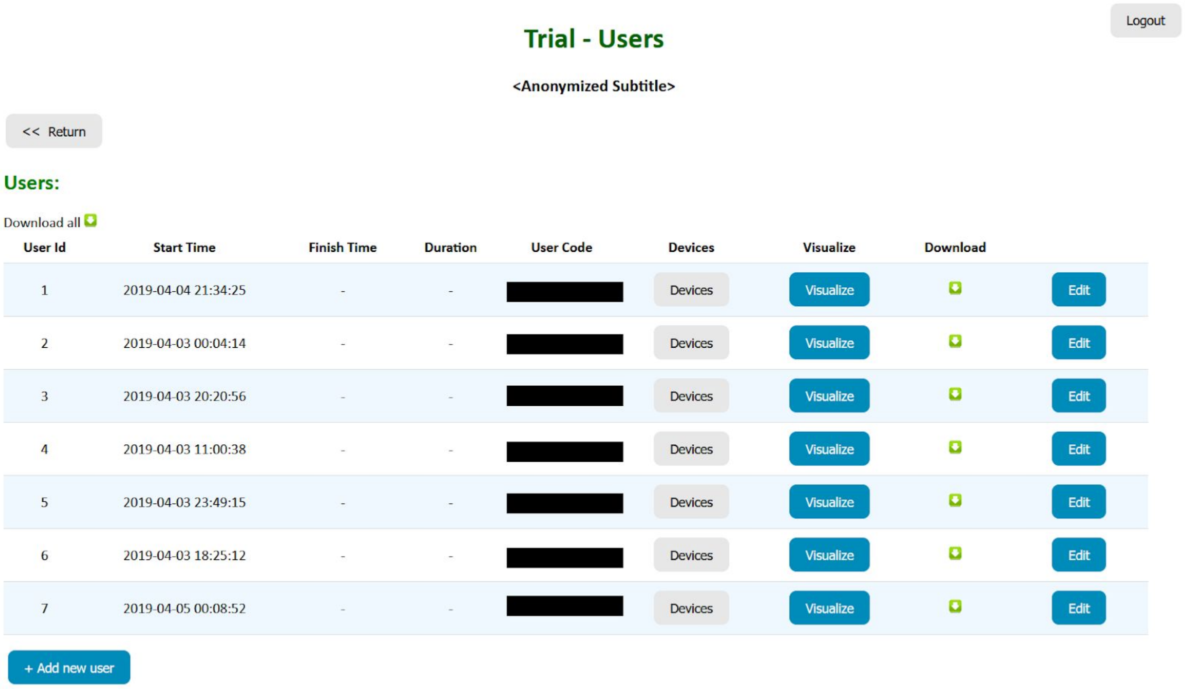

User Id	Start Time	Finish Time	Duration	User Code	Devices	Visualize	Download	
1	2019-04-04 21:34:25	-	-	[redacted]	Devices	Visualize		Edit
2	2019-04-03 00:04:14	-	-	[redacted]	Devices	Visualize		Edit
3	2019-04-03 20:20:56	-	-	[redacted]	Devices	Visualize		Edit
4	2019-04-03 11:00:38	-	-	[redacted]	Devices	Visualize		Edit
5	2019-04-03 23:49:15	-	-	[redacted]	Devices	Visualize		Edit
6	2019-04-03 18:25:12	-	-	[redacted]	Devices	Visualize		Edit
7	2019-04-05 00:08:52	-	-	[redacted]	Devices	Visualize		Edit

Figure 6. - Sample of User's Interface of Trial 1 (user's code were hidden for privacy)

In order to keep track of the existing devices in a project (Edit > Devices) researchers can use the following interface:

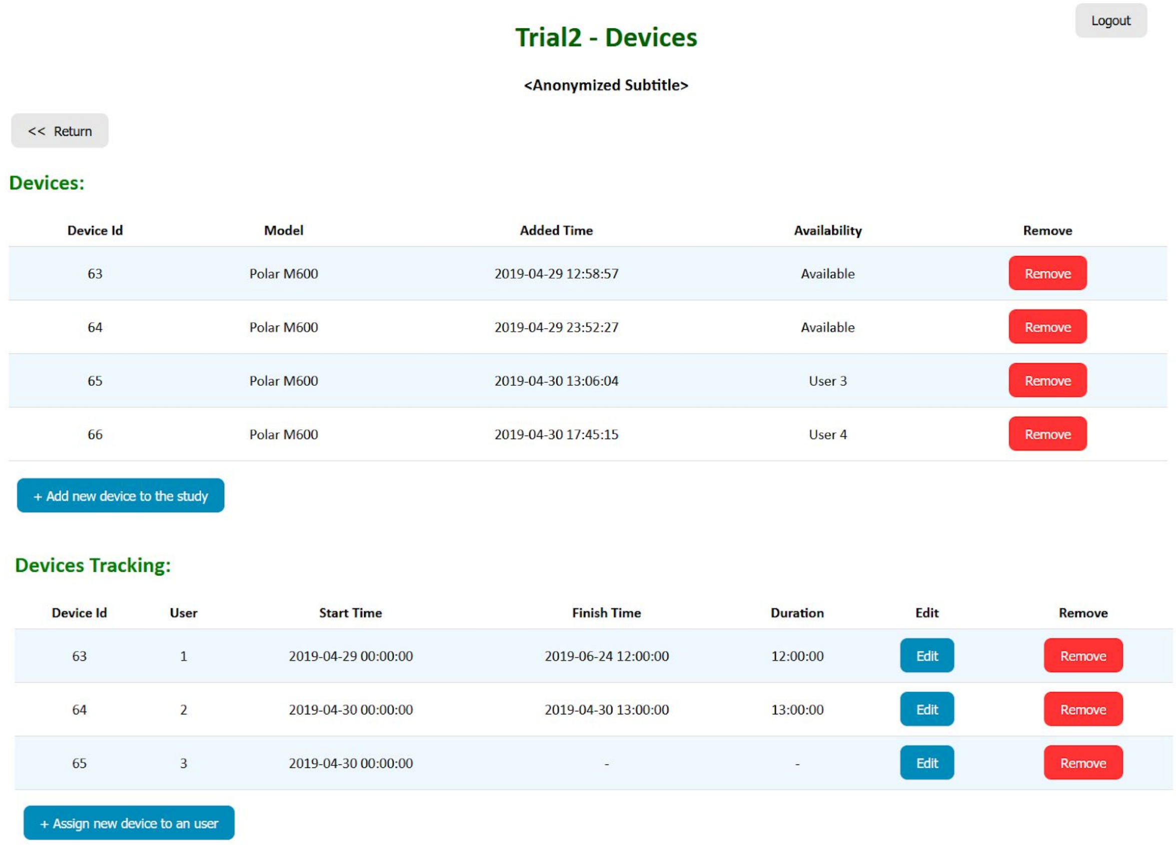

Device Id	Model	Added Time	Availability	Remove
63	Polar M600	2019-04-29 12:58:57	Available	Remove
64	Polar M600	2019-04-29 23:52:27	Available	Remove
65	Polar M600	2019-04-30 13:06:04	User 3	Remove
66	Polar M600	2019-04-30 17:45:15	User 4	Remove

Device Id	User	Start Time	Finish Time	Duration	Edit	Remove
63	1	2019-04-29 00:00:00	2019-06-24 12:00:00	12:00:00	Edit	Remove
64	2	2019-04-30 00:00:00	2019-04-30 13:00:00	13:00:00	Edit	Remove
65	3	2019-04-30 00:00:00	-	-	Edit	Remove

Figure 7. - Sample of Project Device's Interface for Trial 2 project

Sessions data:

A list of all the sessions of a project can be seen in the button ‘Sessions’:

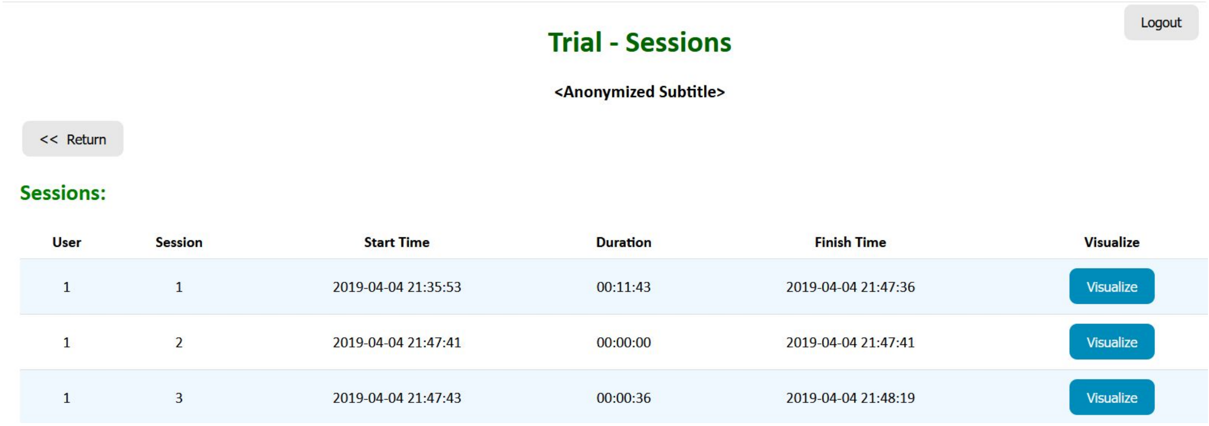

Figure 8. - Sample of sessions interface for Trial 1

Each session’s data (actions, movement and HR) can be seen in detail by clicking on ‘Visualize’:

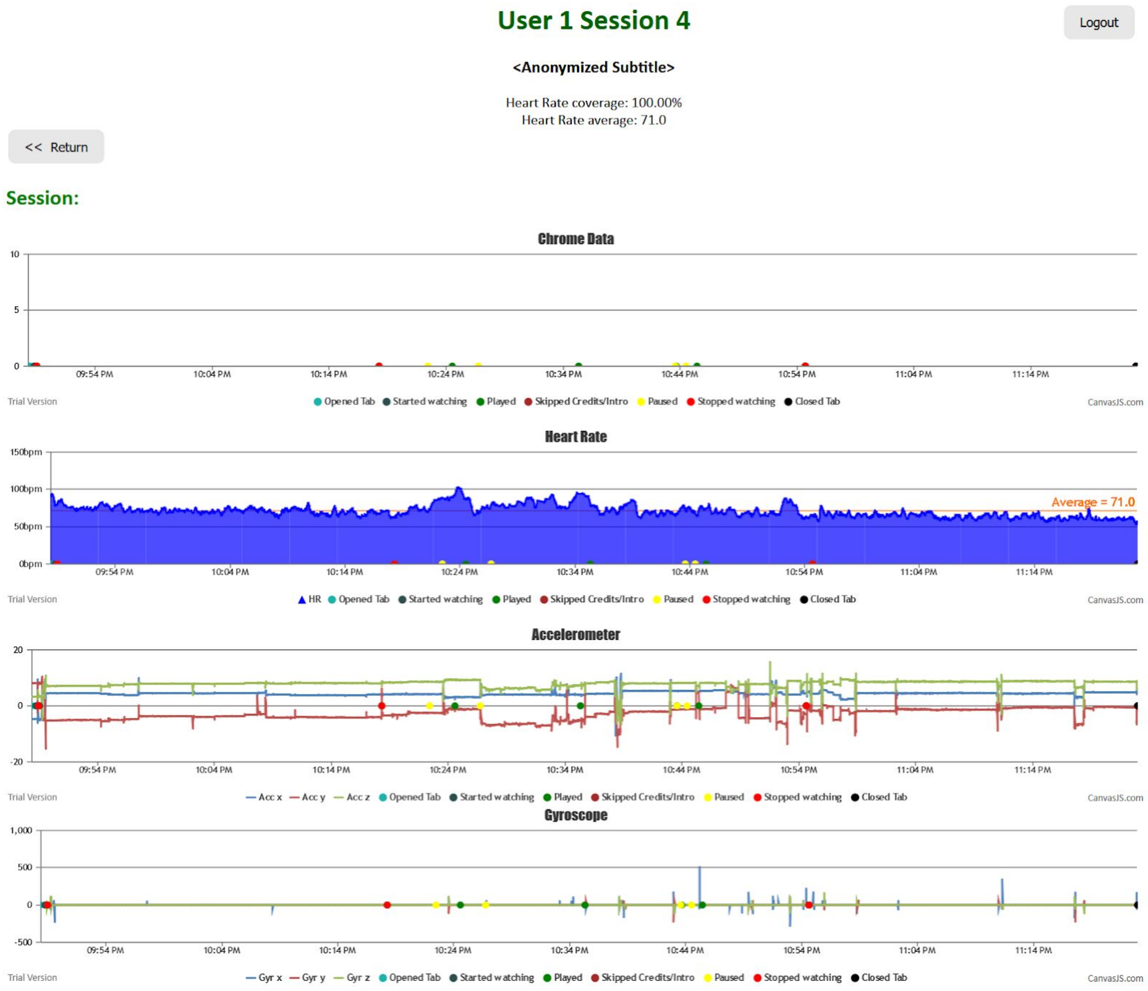

Figure 9. - Session’s data example (Trial 1, user 1, session 4)

At the end of the page, there will be two tables, one with the actions, and other with the HR data:

Time	Title	Season	Episode	Episode Time	Action
2019-04-03 20:22:01					Opened Tab
2019-04-03 20:26:23	Como Defender um Assassino	1	11	00:26:04	Started watching
2019-04-03 20:33:21	Como Defender um Assassino	1	11	00:19:06	Paused
2019-04-03 20:35:22	Como Defender um Assassino	1	11	00:19:06	Played
2019-04-03 20:53:03	Como Defender um Assassino	1	11	00:01:26	Stopped watching
2019-04-03 20:53:23	Como Defender um Assassino	1	12	00:42:59	Started watching
2019-04-03 20:53:26	Como Defender um Assassino	1	12	00:42:55	Paused
2019-04-03 21:03:55	Como Defender um Assassino	1	12	00:42:55	Stopped watching
2019-04-03 21:06:32					Closed Tab

Figure 10. - Actions sample (Trial 1, user 2, session 1)

Time	Heart Rate	Gyro X	Gyro Y	Gyro Z	Acc X	Acc Y	Acc Z
2019-04-03 20:50:33	0	0	0	0	5.57049	1.82572	7.86521
2019-04-03 20:50:34	0	0	0	0	3.92901	0.674776	9.07837
2019-04-03 20:50:35	0	0	0	0	3.81655	0.72024	9.08315
2019-04-03 20:50:36	0	0	0	0	3.70648	0.607777	9.23869
2019-04-03 20:50:37	0	0	0	0	3.72084	0.495314	9.17647
2019-04-03 20:50:38	0	0	0	0	-0.00957129	2.62493	9.27219
2019-04-03 20:50:39	0	0	0	0	0.277567	2.802	9.40858
2019-04-03 20:50:40	0	0	0	0	3.05803	0.569492	8.88934
2019-04-03 20:50:41	0	0	0	0	3.65384	-2.59861	9.52343
[illegible]	[illegible]	[illegible]	[illegible]	[illegible]	[illegible]	[illegible]	[illegible]

Figure 11. - HR Sample (Trial 1, user 2, session 1)

In the user's interface, it is possible to also see all the sessions of a user in a row (Figure 12).

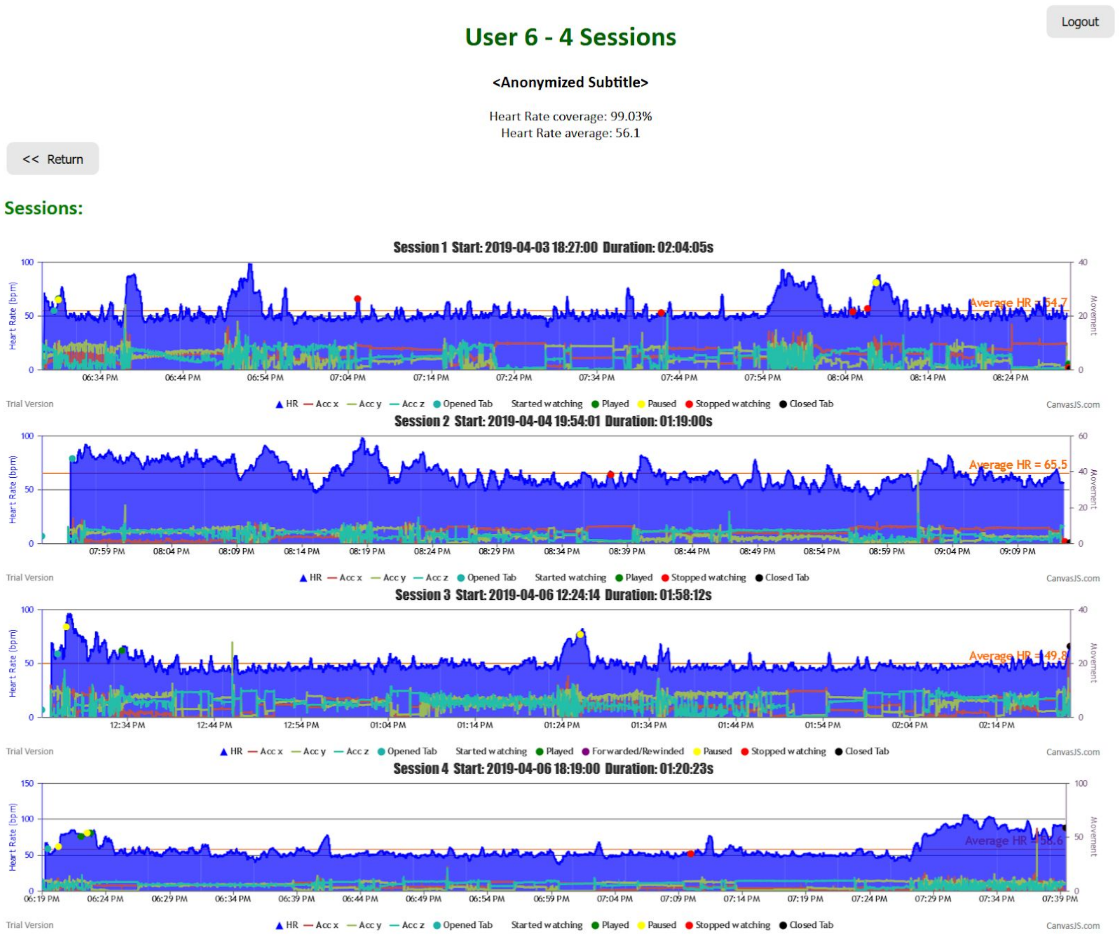

Figure 12. - User's session interface example (Trial 1, user 6)

When mousing over the different graphics, it is possible to read the data of that specific point (actions, HR and movement values) (Figure 13). When the researcher clicks on the graph it's redirected to the detailed page of that session.

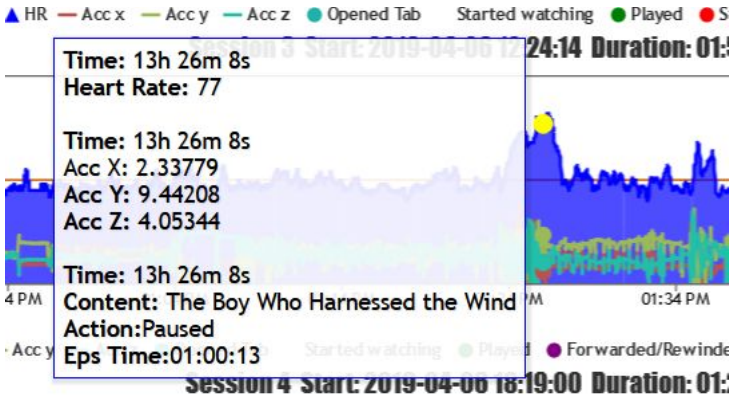

Figure 13. - Mousing over user's session graphic example (Trial 1, user 6)

REPORTS:

In the main interface, when clicking reports of a project, several options will be displayed, making it easy to analyse the data of the project.

The researcher can see all the identified errors of the data of the Chrome Extension and their context in the 'Errors' option of reports (Reports > Errors). See example below:

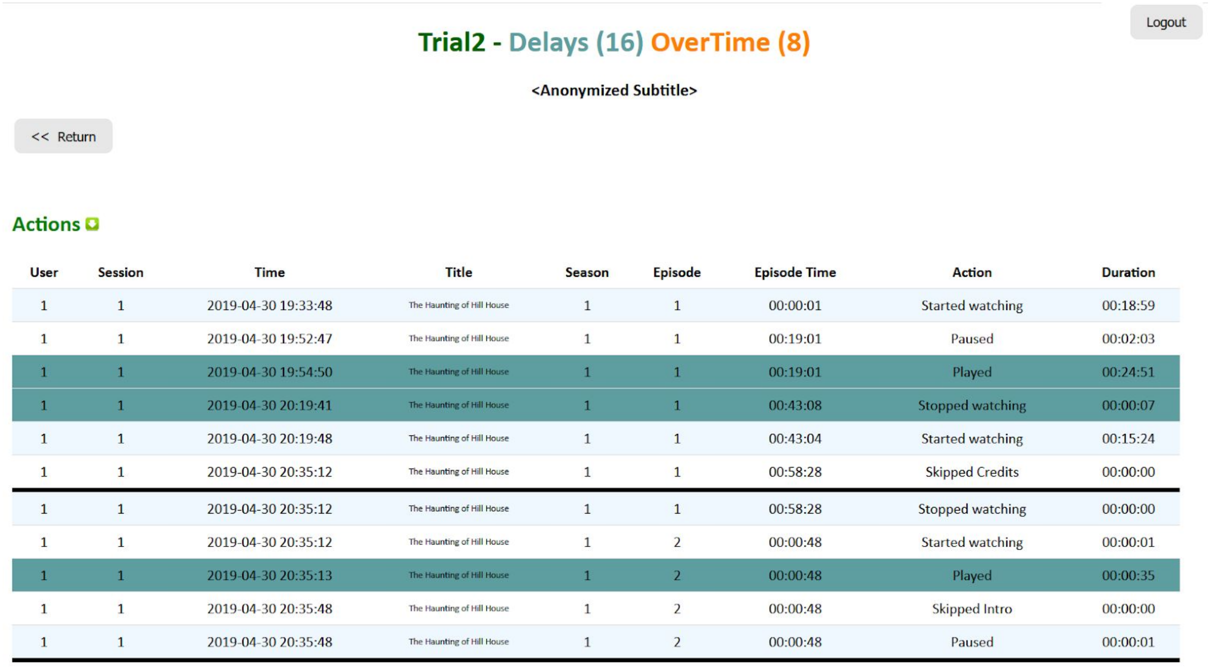
Logout

Trial2 - Delays (16) OverTime (8)

<Anonymized Subtitle>

<< Return

Actions

User	Session	Time	Title	Season	Episode	Episode Time	Action	Duration
1	1	2019-04-30 19:33:48	The Haunting of Hill House	1	1	00:00:01	Started watching	00:18:59
1	1	2019-04-30 19:52:47	The Haunting of Hill House	1	1	00:19:01	Paused	00:02:03
1	1	2019-04-30 19:54:50	The Haunting of Hill House	1	1	00:19:01	Played	00:24:51
1	1	2019-04-30 20:19:41	The Haunting of Hill House	1	1	00:43:08	Stopped watching	00:00:07
1	1	2019-04-30 20:19:48	The Haunting of Hill House	1	1	00:43:04	Started watching	00:15:24
1	1	2019-04-30 20:35:12	The Haunting of Hill House	1	1	00:58:28	Skipped Credits	00:00:00
1	1	2019-04-30 20:35:12	The Haunting of Hill House	1	1	00:58:28	Stopped watching	00:00:00
1	1	2019-04-30 20:35:12	The Haunting of Hill House	1	2	00:00:48	Started watching	00:00:01
1	1	2019-04-30 20:35:13	The Haunting of Hill House	1	2	00:00:48	Played	00:00:35
1	1	2019-04-30 20:35:48	The Haunting of Hill House	1	2	00:00:48	Skipped Intro	00:00:00
1	1	2019-04-30 20:35:48	The Haunting of Hill House	1	2	00:00:48	Paused	00:00:01

Figure 14. - Errors Interface example

It is also possible to see all the actions and the percentage (%) of each episode watched per session (Reports > Actions (view)/(edit)):

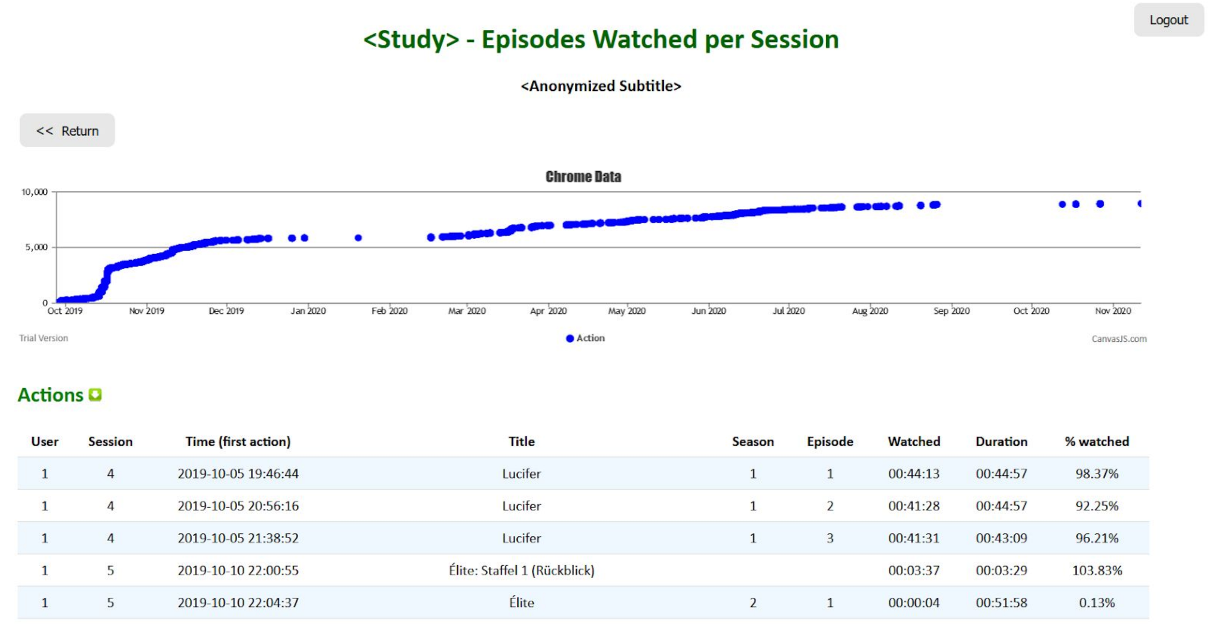

User	Session	Time (first action)	Title	Season	Episode	Watched	Duration	% watched
1	4	2019-10-05 19:46:44	Lucifer	1	1	00:44:13	00:44:57	98.37%
1	4	2019-10-05 20:56:16	Lucifer	1	2	00:41:28	00:44:57	92.25%
1	4	2019-10-05 21:38:52	Lucifer	1	3	00:41:31	00:43:09	96.21%
1	5	2019-10-10 22:00:55	Élite: Staffel 1 (Rückblick)			00:03:37	00:03:29	103.83%
1	5	2019-10-10 22:04:37	Élite	2	1	00:00:04	00:51:58	0.13%

Figure 15. - Example of Actions top Interface

The researcher can also edit some of the actions, keeping the original info, in order to fix some errors, they can occur when transitioning from episode to another. Actions can also be switched since some arrive in the wrong order. repeated or wrong actions can be hidden by checking the box of the column ‘Hidden’ (Reports > Actions (edit)).

User	Session	Time	Title	Season	Eps	Eps Dur	Eps Dur Aux	Eps Code	Eps Time	Eps Time Aux	Action	Duration	Hiden	ID	ID Aux	Move	Dur	Fix
1	0	2019-10-01 21:31:10									Login		☑	14628				
1	1	2019-10-01 21:31:10									Opened Tab	00:00:12	☐	14630				
1	1	2019-10-01 21:31:22									Closed Tab		☐	14640				
1	2	2019-10-01 21:31:24									Opened Tab	00:00:25	☐	14642				
1	2	2019-10-01 21:31:49									Closed Tab		☐	14644				
1	3	2019-10-05 19:20:40									Opened Tab	00:00:01	☐	14772				
1	3	2019-10-05 19:20:41									Login	00:06:12	☐	14774				
1	3	2019-10-05 19:26:53									Closed Tab		☐	14776				

Figure 16. - Example of Actions (edit) bottom Interface

The interface Actions (view) displays all the actions without the editing options (Reports > Actions (view)):

User	Session	Time	Title	Season	Episode	Episode Time	Action	Duration
1	1	2019-10-01 21:31:10					Opened Tab	00:00:12
1	1	2019-10-01 21:31:22					Closed Tab	
1	2	2019-10-01 21:31:24					Opened Tab	00:00:25
1	2	2019-10-01 21:31:49					Closed Tab	
1	3	2019-10-05 19:20:40					Opened Tab	00:00:01
1	3	2019-10-05 19:20:41					Login	00:06:12
1	3	2019-10-05 19:26:53					Closed Tab	
1	4	2019-10-05 19:26:57					Opened Tab	00:19:47
1	4	2019-10-05 19:46:44	Lucifer	1	1	00:00:01	Started watching	00:00:06
1	4	2019-10-05 19:46:50	Lucifer	1	1	00:00:07	Paused	00:00:20
1	4	2019-10-05 19:47:10	Lucifer	1	1	00:00:07	Stopped watching	00:24:59
1	4	2019-10-05 20:12:09	Lucifer	1	1	00:00:06	Started watching	00:44:07
1	4	2019-10-05 20:56:16	Lucifer	1	1	00:44:13	Stopped watching	00:00:00
1	4	2019-10-05 20:56:16	Lucifer	1	2	00:01:29	Started watching	00:00:03
1	4	2019-10-05 20:56:19	Lucifer	1	2	00:01:29	Played	00:00:14

Figure 17. - Example of Actions (view) bottom Interface

For all the sessions with HR coverage, it is possible to see where HR data is missing (Reports > HR coverage faults):

Trial2 - Data Failures

Logout

<Anonymized Subtitle>

<< Return

HR Gaps:

User	Session	Init	End	Start	Duration	Finish
1	2	2019-05-03 19:32:48	2019-05-03 19:32:48	2019-05-03 19:32:48	00:00:00	2019-05-03 19:32:48
2	1	2019-05-05 16:29:12	2019-05-05 17:33:32	2019-05-05 16:29:12	01:04:20	2019-05-05 17:33:32
3	1	2019-04-30 18:38:17	2019-04-30 18:38:38	2019-04-30 18:38:17	00:00:21	2019-04-30 18:38:38
3	2	2019-04-30 18:38:38	2019-04-30 18:42:10	2019-04-30 18:38:38	00:03:02	2019-04-30 18:41:40
3	6	2019-04-30 18:42:56	2019-04-30 19:58:08	2019-04-30 19:52:15	00:05:53	2019-04-30 19:58:08

Figure 18. - HR gaps in each session

It is also possible to visualize all the sessions HR coverage as a table or in an histogram (Reports > Sessions HR coverage) (Figure 19). The sessions are classified as Empty, Valid or Semi-Valid (Semi_valid: when no closing tab action is found).

Trial2 - Sessions Coverage

Logout

<Anonymized Subtitle>

<< Return

Sessions:

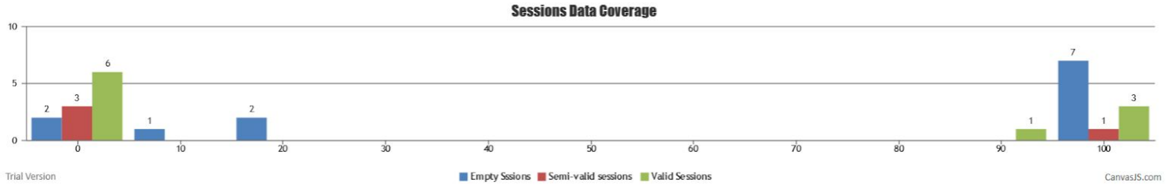

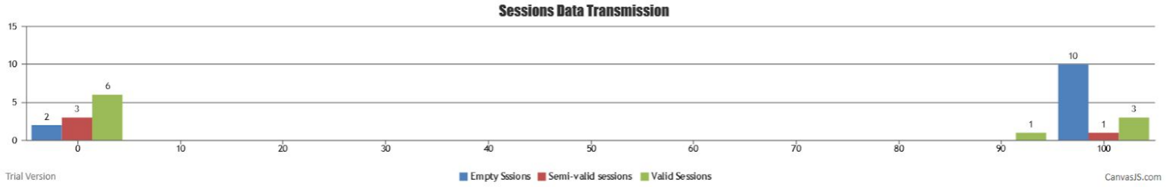

User	Session	Category	Start	Duration	End	Data Coverage	Data Transmission
1	1	Valid	2019-04-30 19:32:44	01:43:23	2019-04-30 21:16:07	100.00%	100.00%
1	2	Empty	2019-05-03 19:32:48	00:00:00	2019-05-03 19:32:48	0.00%	0.00%
2	1	Valid	2019-05-05 16:29:12	01:04:20	2019-05-05 17:33:32	0.00%	0.00%
3	1	Empty	2019-04-30 18:38:17	00:00:21	2019-04-30 18:38:38	0.00%	0.00%
3	2	Empty	2019-04-30 18:38:38	00:03:32	2019-04-30 18:42:10	14.08%	100.00%
3	3	Empty	2019-04-30 18:42:11	00:00:19	2019-04-30 18:42:30	100.00%	100.00%

Figure 19. - HR coverage for each session

A list of how many participants watched each episode can be found on Reports > Contents:

Logout

Trial2 - Contents

<Anonymized Subtitle>

<< Return

Contents:

Title	Season	Episode	Users	Episode Duration	Visualize
Paradise Police	1	1	1	01:29:30	Visualize
Paradise Police	1	2	1	01:27:11	Visualize
The Asterisk War	1	24	1	01:23:40	Visualize
The Haunting of Hill House	1	1	6	02:00:06	Visualize
The Haunting of Hill House	1	2	2	01:51:17	Visualize

Figure 20. - How many users watched each content in a Study
